# The walnut transcription factor *JrGRAS2* contributes to high temperature stress tolerance involving in Dof transcriptional regulation and HSP protein expression

**DOI:** 10.1186/s12870-018-1568-y

**Published:** 2018-12-20

**Authors:** Guiyan Yang, Xiangqian Gao, Kaiheng Ma, Dapei Li, Caixia Jia, Meizhi Zhai, Zhenggang Xu

**Affiliations:** 1 Laboratory of Walnut Research Center, College of Forestry, Northwest A & F University, Yangling, 712100 Shaanxi China; 2 Key Laboratory of Economic Plant Resources Development and Utilization in Shaanxi Province, College of Forestry, Northwest A & F University, Yangling, 712100 Shaanxi China; 3 Hunan Research Center of Engineering Technology for Utilization of Environmental and Resources Plant, Central South University of Forestry and Technology, 498 Shaoshan South Road, Changsha, 410004 Hunan Province China

**Keywords:** Transcriptional regulation, Promoter, Dof transcription factor, GRAS transcription factor, High temperature stress

## Abstract

**Background:**

GRAS transcription factor (TF) family is unique and numerous in higher plants with diverse functions that involving in plant growth and development processes, such as gibberellin (GA) signal transduction, root development, root nodule formation, and mycorrhiza formation. Walnut tree is exposed to various environmental stimulus that causing concern about its resistance mechanism. In order to understand the molecular mechanism of walnut to adversity response, a GRAS TF (*JrGRAS2*) was cloned and characterized from *Juglans regia* in this study.

**Results:**

A 1500 bp promoter fragment of *JrGRAS2* was identified from the genome of *J. regia*, in which the *cis*-elements were screened. This *JrGRAS2* promoter displayed expression activity that was enhanced significantly by high temperature (HT) stress. Yeast one-hybrid assay, transient expression and chromatin immunoprecipitation (Chip)-PCR analysis revealed that *JrDof3* could specifically bind to the DOFCOREZM motif and share similar expression patterns with *JrGRAS2* under HT stress. The transcription of *JrGRAS2* was induced by HT stress and up-regulated to 6.73-~11.96-fold in the leaf and 2.53-~4.50-fold in the root to control, respectively. *JrGRAS2* was overexpressed in *Arabidopsis*, three lines with much high expression level of *JrGRAS2* (S3, S7, and S8) were selected for HT stress tolerance analysis. Compared to the wild type (WT) *Arabidopsis*, S3, S7, and S8 exhibited enhanced seed germination rate, fresh weight accumulation, and activities of catalase (CAT), peroxidase (POD), superoxide dismutase (SOD) and glutathione-S-transferase (GST) under HT stress. In contrast, the Evans blue staining, electrolyte leakage (EL) rates, hydrogen dioxide (H_2_O_2_) and malondialdehyde (MDA) content of transgenic seedlings were all lower than those of WT exposed to HT stress. Furthermore, the expression of heat shock proteins (HSPs) in S3, S7, and S8 was significant higher than those in WT plants. The similar results were obtained in *JrGRAS2* transient overexpression walnut lines under normal and HT stress conditions.

**Conclusions:**

Our results suggested that JrDof3 TF contributes to improve the HT stress response of *JrGRAS2*, which could effectively control the expression of *HSP*s to enhance HT stress tolerance. *JrGRAS2* is an useful candidate gene for heat response in plant molecular breeding.

**Electronic supplementary material:**

The online version of this article (10.1186/s12870-018-1568-y) contains supplementary material, which is available to authorized users.

## Background

High temperature (HT) stress is one of the most important limiting factors to plant growth and productivity [1]; warming surface temperatures and increasing frequency and duration of widespread droughts threaten the health of natural forests and agricultural crops [2]. The rising temperature may cause a change in the growing periods and the distribution of plants. HT may inactivate major enzymes, disturb protein synthesis, damage proteins and membranes, have major effects on the process of cell divisions, all of these can favor the oxidative damage and seriously limit the plant growth [1, 3]. Other than this, long-term HT stress during the seed filling can result in poor quality and low yield [4–6]. For instance, the number of spikes and florets per plant in rice and the seed-set in sorghum were negatively affected by HT stress [7, 8]. Under high night temperature, a decrease in individual grain weight resulted in significant reduction in rice grain production per unit area [9]. Zhao et al. (2017) point out that without effective adaptation, CO_2_ fertilization, and genetic improvement, each degree-Celsius increase in global mean temperature would, on average, reduce global yields of maize by 7.4%, wheat by 6.0%, rice by 3.2%, and soybean by 3.1% [10]. Therefore, the damage caused by HT to plants should not be underestimated.

The effect of HT varies in different plant species and cultivars, and even at different developmental stages within a species. To enable the production of plants with improved thermotolerance, decoding the mechanisms that which plants cope with HT is very necessary [11]. In recent years, physiological, biochemical, genetic, and molecular studies have revealed a number of vital cellular components and processes involved in thermoresponsive growth and the acquisition of thermotolerance in plants [11]. During these processes, a series of genes are employed that including heat shock proteins (HSPs) and reactive oxygen species (ROS)-scavenging enzymes, which were classified into two groups as follows: (1) The genes involve in heat shock signaling mechanisms that mainly including HSFA1-dependent transcriptional regulation networks, HSFA1-independent transcription regulation networks, Calcium (Ca^2+^) signaling, ROS signaling, NO signaling, Hydrogen sulfide (H_2_S) signaling, and unfolded protein response (UPR) [11–20]. (2) The genes associate with high ambient temperature signaling mechanisms, which contain the coordinated regulation of circadian clock [21], phytohormone signaling [22], and light signaling [23]. The core of these various signaling pathways is partially integrated to the basic helix-loop-helix (bHLH) transcription factor (TF) phytochrome interacting factor 4 (PIF4) [11, 21, 22]. PIF4 is connected with abundant genes such as: ultraviolet (UV) resistance locus 8 (*UVR8*) [24], constitutively photomorphogenic 1 (*COP1*) [25], elongated hypocotyl 5 (*HY5*) [26]. In PIF4-dependent ambient HT responses, PIF4 can activate the expression of auxin biosynthesis-related genes such as cytochrome *P450*, YUCCA 8 (*YUC8*), and tryptophan aminotransferase of *Arabidopsis* 1 (*TAA1*) via binding to their promoters [27, 28]; PIF4 can synergistically promote the transcription of genes required for hypocotyl elongation such as auxin response factor 6 (*ARF6*) [29]; In addition, PIF4 can integrate brassinosteroid (BR) and gibberellin (GA) signaling by interacting directly with their central components [29, 30].

In GA signaling, the GRAS TF family is one of the important members, which is unique to higher plants and discovered in recent years. The name of GRAS is derived from the three initially identified members, GA insensitive (GAI), repressor of GA1 (RGA) and scarecrow (SCR) [31]. In addition to play the role in GA signal transduction, studies have demonstrated that GRAS TFs play diverse roles in light signaling, root and meristem development, biotic and abiotic stress responses [32]. According to functional differences and structural characteristics, GRAS TFs were divided into several sub-families such as DELLA, SCR, SHR, SCL3, PAT, and LISCL [33]. Among which, the SCL proteins were considered as members of HT stress response signal pathway. For instance, the levels of cabbage GRAS TF *BoSCL13* was increased with heat shock and confirmed as a unique candidate gene for discriminating heat shock tolerance in cabbage breeding [34]. The transcription of *AtSCL13* (At4g17230) was increased by heat shock at an early time point after heat treatment [34]. However, the reports on GRAS to HT stress are few, future studies on the specific roles of GRAS TFs in heat tolerance and/or heat response is necessary.

*Juglans regia* is a nut tree cultivated worldwide and famous for its nutritious fruits [35]. As in all other plant species, walnut tree is sessile and cannot escape the unfavorable environmental conditions [36]. The growth, development, and production of *J. regia* are all affected by environmental stimulus, such as: high temperature, cold, salinity, and drought stress [37, 38]. However, studies on the stress response mechanism of walnut trees are currently lacking; achieving a better understanding of the mechanisms involved in abiotic stress response of *J. regia* is timely and essential [39]. In previous studies, we identified a few candidate genes from walnut tree relating to stress response, including some members of GRAS TF family, among which a SCL protein (Named as *JrGRAS2*) was detected to be induced by HT, and could improve the heat tolerance of yeast [40]. In this study, we further explore the function mechanism of *JrGRAS2* response to HT stress, and found *JrGRAS2* is a positive factor for plant HT tolerance associating with Dof TF and HSP protein.

## Results

### Identification and HT stress response of *JrGRAS2* promoter

**Fig. 1:**
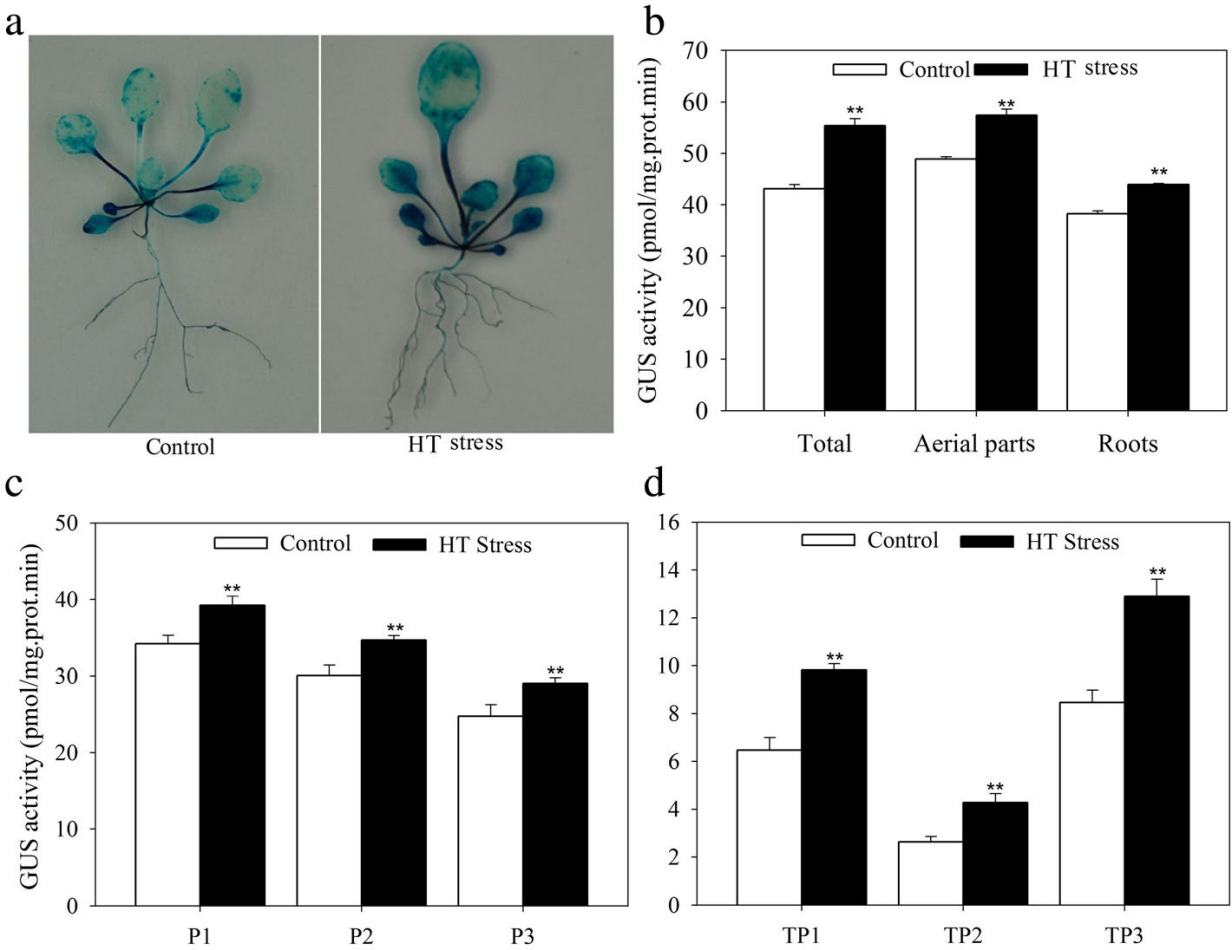
The expression activity of *JrGRAS2* promoter under normal and HT stress. The significant differences between the HT stress and normal conditions were marked as two asterisk (**) (*p*<0.01). **a**, GUS staining of the *JrGRAS2* promoter transformed *Arabidopsis* plants under normal and HT treatments. **b**, the GUS activities according to A. **c**, The GUS activities of three transgenic lines that transformed by *JrGRAS2* promoter (P1, P2, P3) under normal and HT stress. **d**, The GUS activities of *JrGRAS2* promoter transient expressed walnut leaves under normal and HT stress. TP1, TP2, TP3, three transient expression of *JrGRAS2* promoter walnut lines

A 1500 bp promoter segment of *JrGRAS2* was identified from the *J. regia* genome that was located in the 874811-870000 (NW_017443591.1) region of the walnut genome [41]. This promoter contains abundant *cis*-elements which are grouped into some classes, such as class of ‘ABA, Dehydration & salinity (osmotic) stress responsive’ includes the motifs of ABRE, MYB, DRE; class of ‘Miscellaneous’ covers the motifs of DOFCOREZM, RAV1AAT, SEF1MOTIF, POLASIG3, and so on (Additional file 1: Figure S1, Additional file 2: Table S1). The *JrGRAS2* promoter fragment was inserted into pCAMBIA1301 vector and then transformed into *Arabidopsis* and walnut plants, which were further stained to reveal that the promoter caused *GUS* expression in the leaves and roots. Meanwhile, comparing to control, the GUS activities of *JrGRAS2* promoter transgenic plants were significantly induced by HT stress (Fig. 1).

### *JrDof3* acts as the up-stream regulator of *JrGRAS2* in HT stress response

**Fig. 2:**
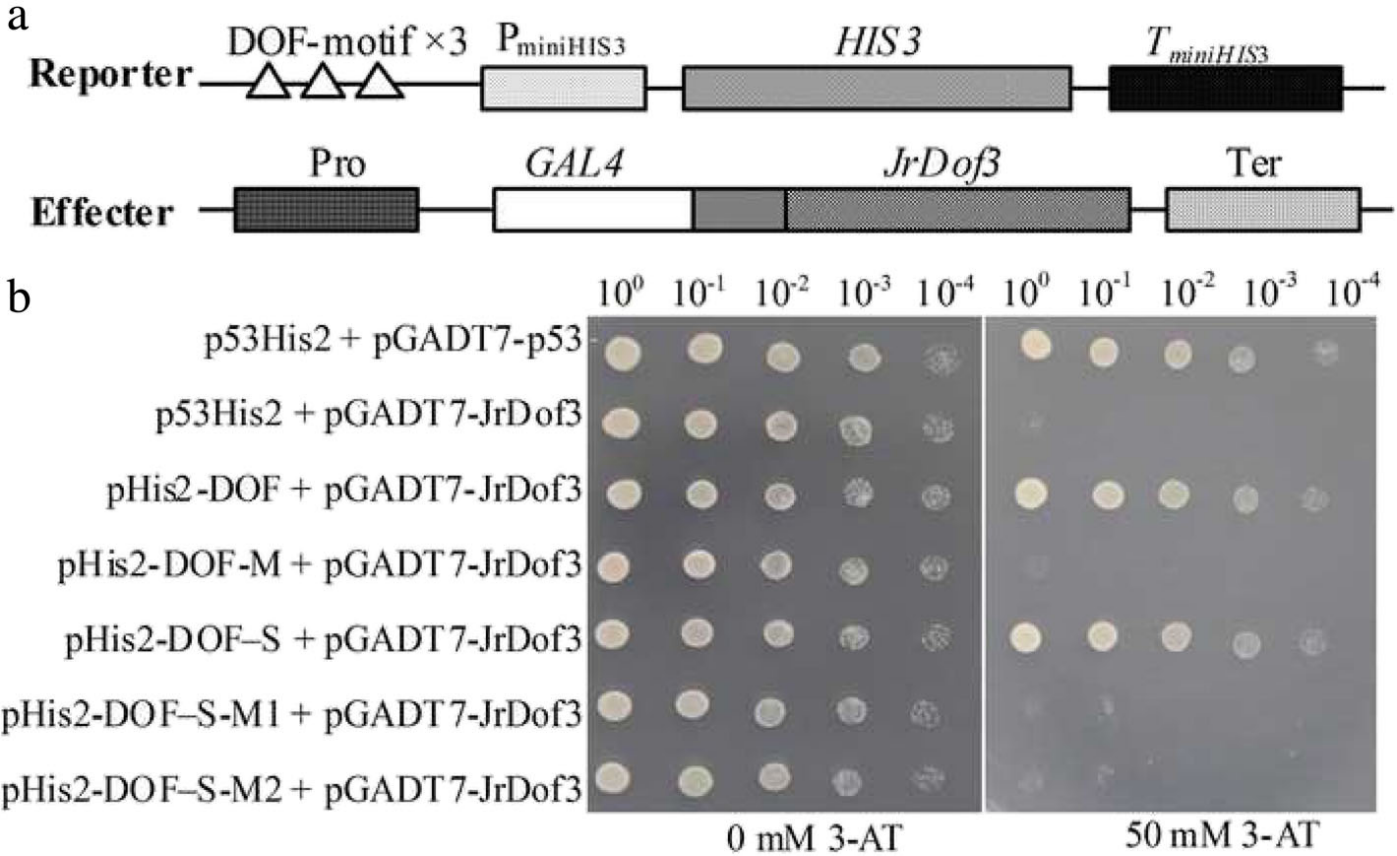
Identification of the up-steam regulator of *JrGRAS2* using yeast one-hybrid assays. **a**, Diagram of the reporter and effecter vectors. Three tandem copies of the DOFCOREZM motif were inserted into the pHis2 vector and used as the reporter construct. The CDS of *JrDof3* was cloned into pGADT7-Rec2 and used as the effecter construct. **b**, The effecter and reporter constructs were co-transformed into the yeast strain Y187. p53His2 + pGADT7-p53, positive control, pGADT7-Rec2 vector that encodes murine p53 fused with GAL4 AD; p53His2 + pGADT7-JrDof3, negative control, pHis2 reporter vector that contains the *cis*-acting DNA consensus sequence recognized by p53. pHis2-DOF + pGADT7-JrDof3, pHis2-DOF-M + pGADT7-JrDof3, pHis2-DOF-S + pGADT7-JrDof3, pHis2-DOF-M1 + pGADT7-JrDof3, pHis2-DOF-M2 + pGADT7-JrDof3 means the interaction DOFCOREZM motif, mutated DOFCOREZM motif, promoter segment including the DOFCOREZM motif, promoter segment containing the mutated DOFCOREZM motif, promoter segment excluding the DOFCOREZM motif with JrDof3, accordingly. The transformants spotted on TDO plates with 0 mM 3-AT were used as positive controls for transformants growth. Positive transformants were further confirmed by spotting serial dilutions (1/1, 1/10, 1/100, 1/1000, 1/10000) onto TDO plates with 50 mM 3-AT

To screen the up-stream regulator of *JrGRAS2*, the *cis*-elements distributed in the promoter were analyzed and found that DOFCOREZM motif is the most abundant one (Additional file 2: Table S1). Considering the diverse function of Dof TFs in plant stress response, yeast one-hybrid assays were employed to study the interactions between Dof TFs and DOFCOREZM in the promoter. The results showed that JrDof3 could bind to DOFCOREZM motif, which was verified by the interactions between JrDof3 and the mutated DOFCOREZM motif (pHis2-DOF-M), or promoter segment including the DOFCOREZM motif (pHis2-DOF-S), or promoter segment containing the mutated DOFCOREZM motif (pHis2-DOF-M1), or promoter segment excluding the DOFCOREZM motif (pHis2-DOF-M2) on the solid synthetic drop-out medium (SD)/-Trp-Leu-His plus with 50 mM 3-amino-1, 2, 4-triazole (3-AT) (Fig. 2).

**Fig. 3:**
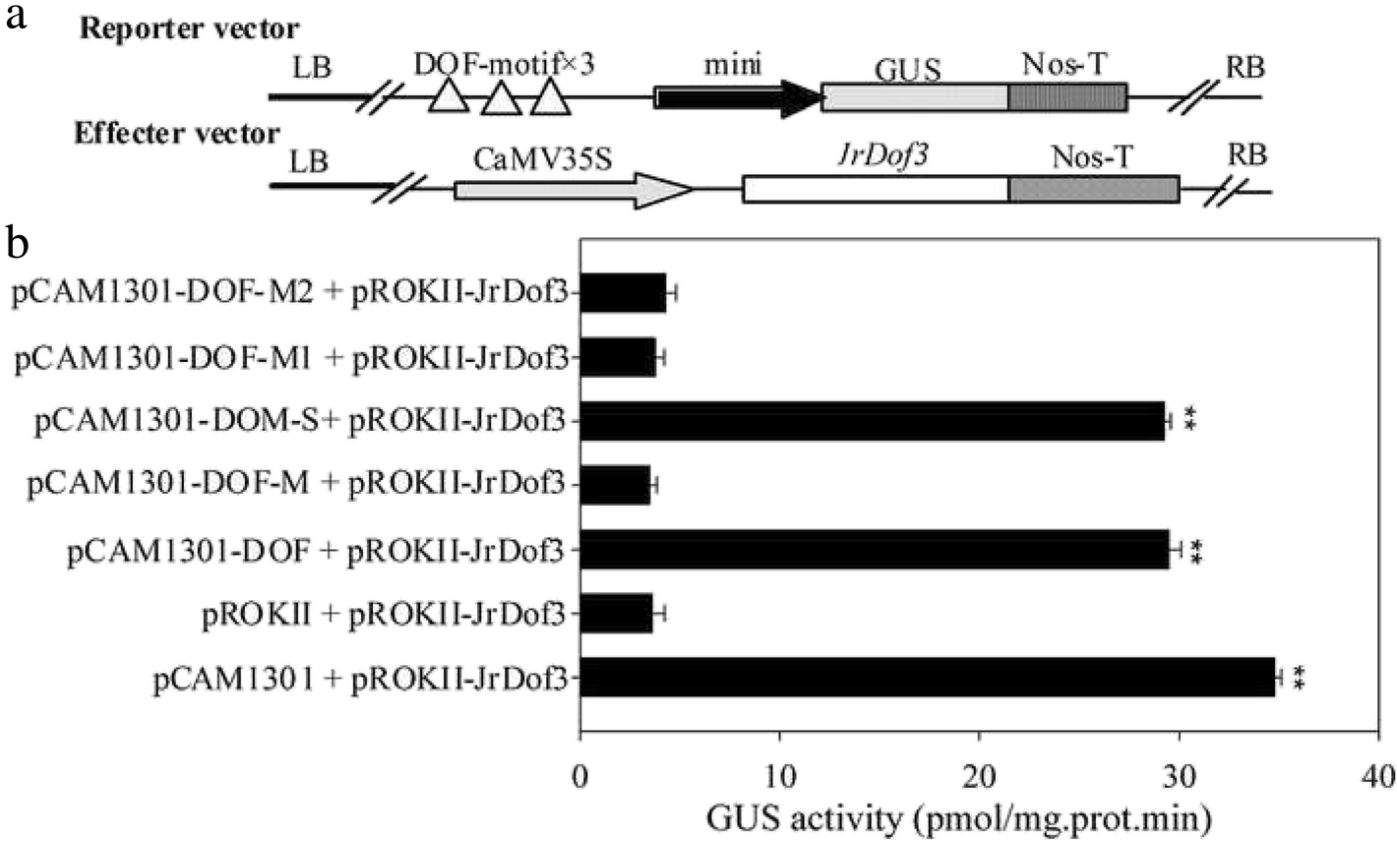
Transient co-expression analysis of the interactions obtaining from the yeast one-hybrid assays. **a**, Diagram of the reporters and effecters. Triple tandem copies of the DOFCOREZM motif were fused with the 35S CaMV-46 minimal promoter and cloned into pCAMBIA1301 for driving the *GUS* gene which used as the reporter construct. The CDS of *JrDof3* was cloned into pROKII under the control of the 35S promoter and used as the effecter construct. **b**, The GUS activities of the transformed tobacco seedlings were determined. pCAM1301 + pROKII-JrDof3, positive control, pCAMA1301 vector transformed with JrDof3; pROKII + pROKII-JrDof3, negative control, pROKII vector transformed with JrDof3. pCAM1301-DOF + pROKII-JrDof3, pCAM1301-DOF-M + pROKII-JrDof3, pCAM1301-DOF-S + pROKII-JrDof3, pCAM1301-DOF-M1 + pROKII-JrDof3, pCAM1301-DOF-M2 + pROKII-JrDof3, represented the transformation of reporter DOFCOREZM motif, mutated DOFCOREZM motif, promoter segment including the DOFCOREZM motif, promoter segment containing the mutated DOFCOREZM motif, promoter segment excluding the DOFCOREZM motif transformed with the effecter, accordingly. Two asterisk means significant differences (*p*<0.01) between the negative control and others

**Fig. 4:**
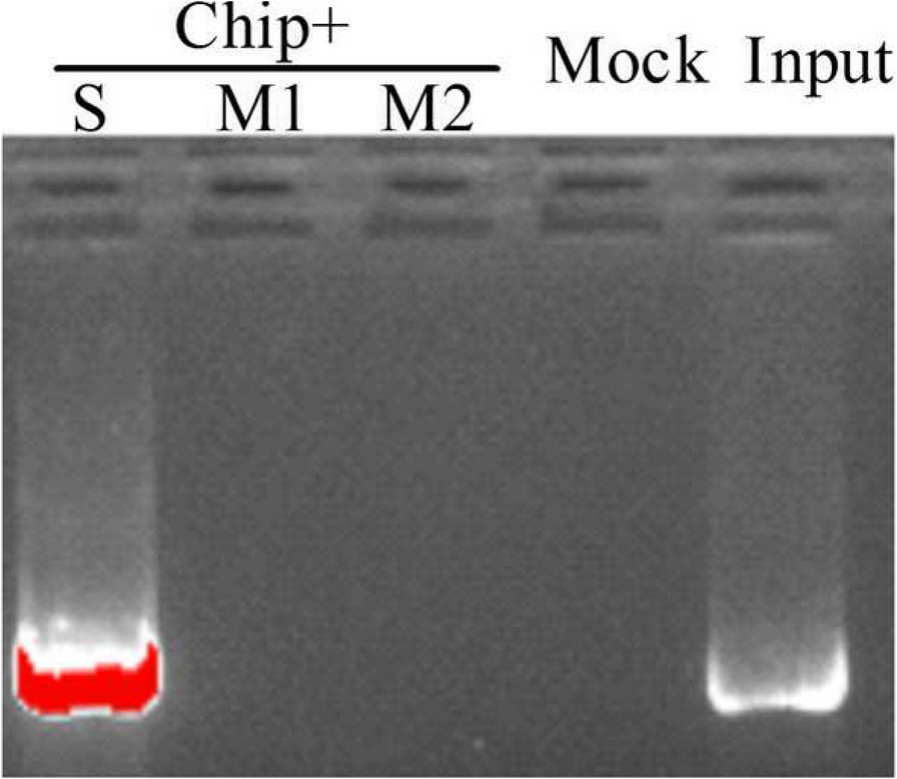
Chip-PCR analysis of direct binding of JrDof3 to *JrGRAS2* promoter. S, the promoter segment including the DOFCOREZM motif; M1, promoter segment containing the mutated DOFCOREZM motif; M2, promoter segment excluding the DOFCOREZM motif; Input, positive control; Mock, negative control

The special binding of JrDof3 to DOFCOREZM motif was confirmed by co-transformation of the reporter --DOFCOREZM motif (pCAM1301-DOF), or mutated DOFCOREZM motif (pCAM1301-DOF-M), or promoter segment including the DOFCOREZM motif (pCAM1301-DOF-S), or promoter segment containing the mutated DOFCOREZM motif (pCAM1301-DOF-M1), or promoter segment excluding the DOFCOREZM motif (pCAM1301-DOF-M2) with the effecter (pROKII-*JrDof3*) (Fig. 3a), which showed that the GUS activities of the leaves transformed by DOFCOREZM motif or promoter segment including the DOFCOREZM motif were similar to the positive controls and significant higher than those of the negative control and mutated reporters (Fig. 3b). Moreover, the promoter segment including the DOFCOREZM motif (S), or promoter segment containing the mutated DOFCOREZM motif (M1), or promoter segment excluding the DOFCOREZM motif (M2) was chosen for Chip-PCR to analyze the direct binding of JrDof3 to the *JrGRAS2* promoter, and Chip+S displayed similar gel electrophoresis strip with input (positive control), while M1 and M2 showed similar results with mock (negative control) (Fig. 4), suggesting that JrDof3 could directly bind to *JrGRAS2* promoter and function as an up-stream regulator of *JrGRAS2*.

**Fig. 5:**
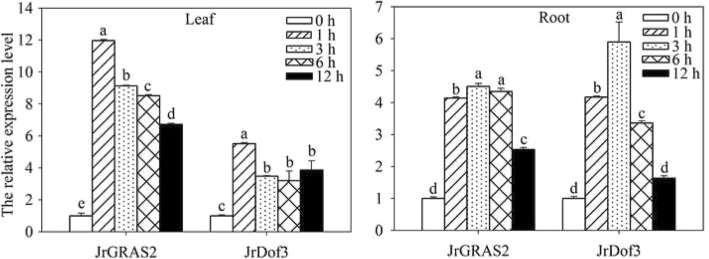
The expression of *JrGRAS2* and *JrDof3* in *J. regia* leaf and root tissue under HT stress. The relative expression level = transcription level under stress treatment/transcription level under control condition. Error bars were obtained from three replicates of qRT-PCR. The lowercase a-e mean the significant differences among the treatment time points (*p*<0.05)

Furthermore, quantitative real-time PCR (qRT-PCR) analysis of the expression of *JrDof3* and *JrGRAS2* response to HT stress displayed that both of *JrDof3* and *JrGRAS2* were induced by HT with leaf and root tissue specificity (Fig. 5). In the leaves, the transcription levels of *JrDof3* and *JrGRAS2* were similar and declined from 1 to 6 h, the expression of *JrGRAS2* was 6.73-~11.96-fold and the expression of *JrDof3* was 3.20-~5.50-fold of control exposed to 1~ 12 h stress of HT, accordingly. In the roots, *JrDof3* and *JrGRAS2* showed same expression patterns that they were increased from 1 to 3 h then decreased from 3 to 12 h. The maximum transcription values of *JrDof3* and *JrGRAS2* were 5.90- and 4.50-fold of control, respectively (Fig. 5). The similar induction of *JrDof3* and *JrGRAS2* by HT indicated that *JrDof3* act as an up-stream regulator of *JrGRAS2* in HT stress response.

### Overexpression of *JrGRAS2* up-regulated plant HT stress tolerance

**Fig. 6:**
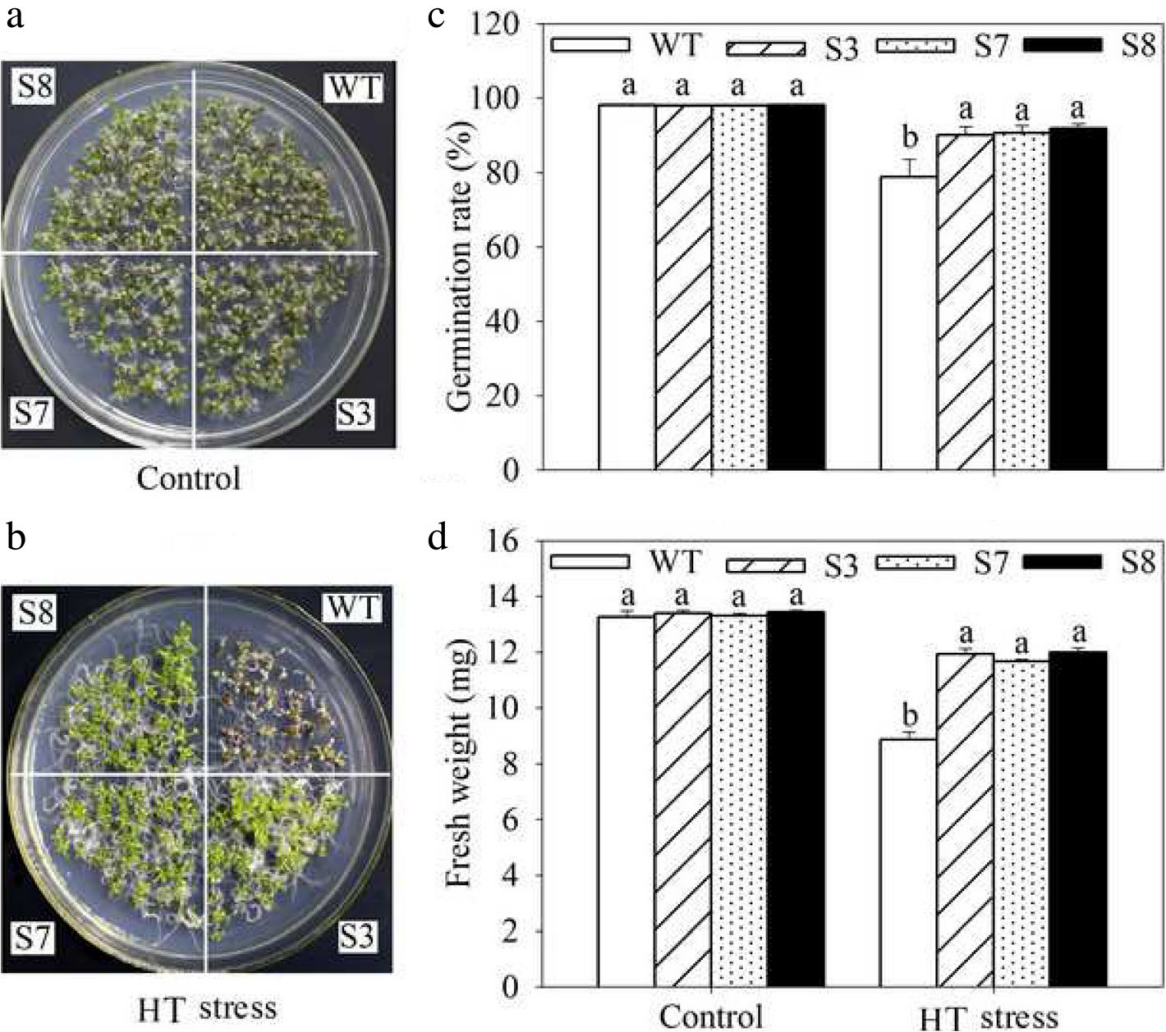
The germination and growth of *JrGRAS2* transgenic plants exposed to high temperature (HT) stress comparing with wild type (WT) *Arabidopsis*. S3, S7 and S8 were three *JrGRAS2* transgenic lines. **a**-**b**, germination under normal and HT conditions that sown on the 1/2MS agar medium for 12 d. **c**, germination percentage of WT, S3, S7 and S8 according to **a** and **b**. **d**, average fresh weight of the germinated seedlings from **a** and **b**. The significant differences between WT and transgenic lines were marked with lowercase (*p*<0.05)

**Fig. 7:**
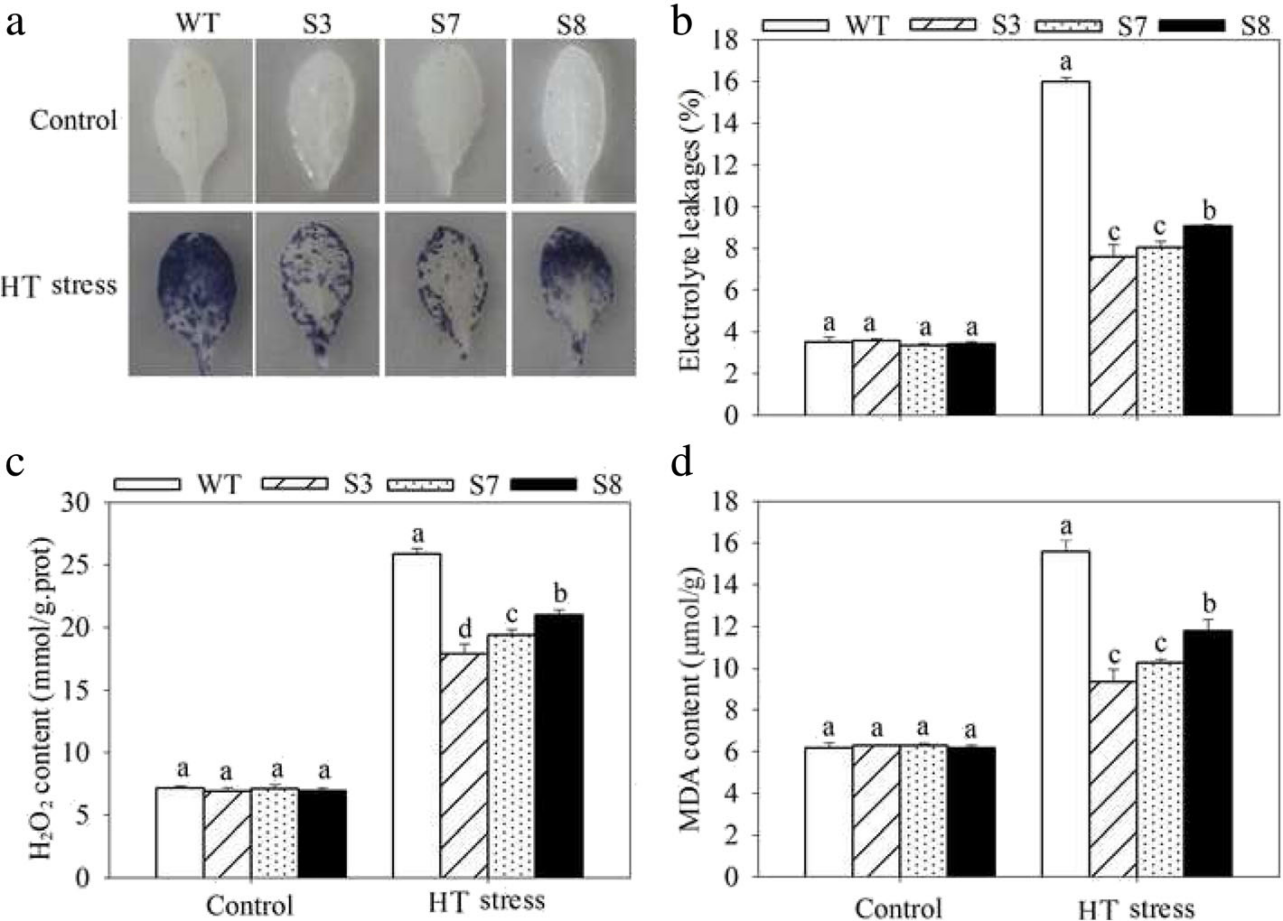
The ROS accumulation in transgenic and WT plants under HT stress. Five-week-old seedlings were treated with HT stress for one-week and used for analysis. **a**, Evans blue staining; **b**, EL rate; **c**, H_2_O_2_ content; **d**, MDA content. The significant differences (*p*<0.05) between transgenic lines and WT were indicated by lowercase

**Fig. 8:**
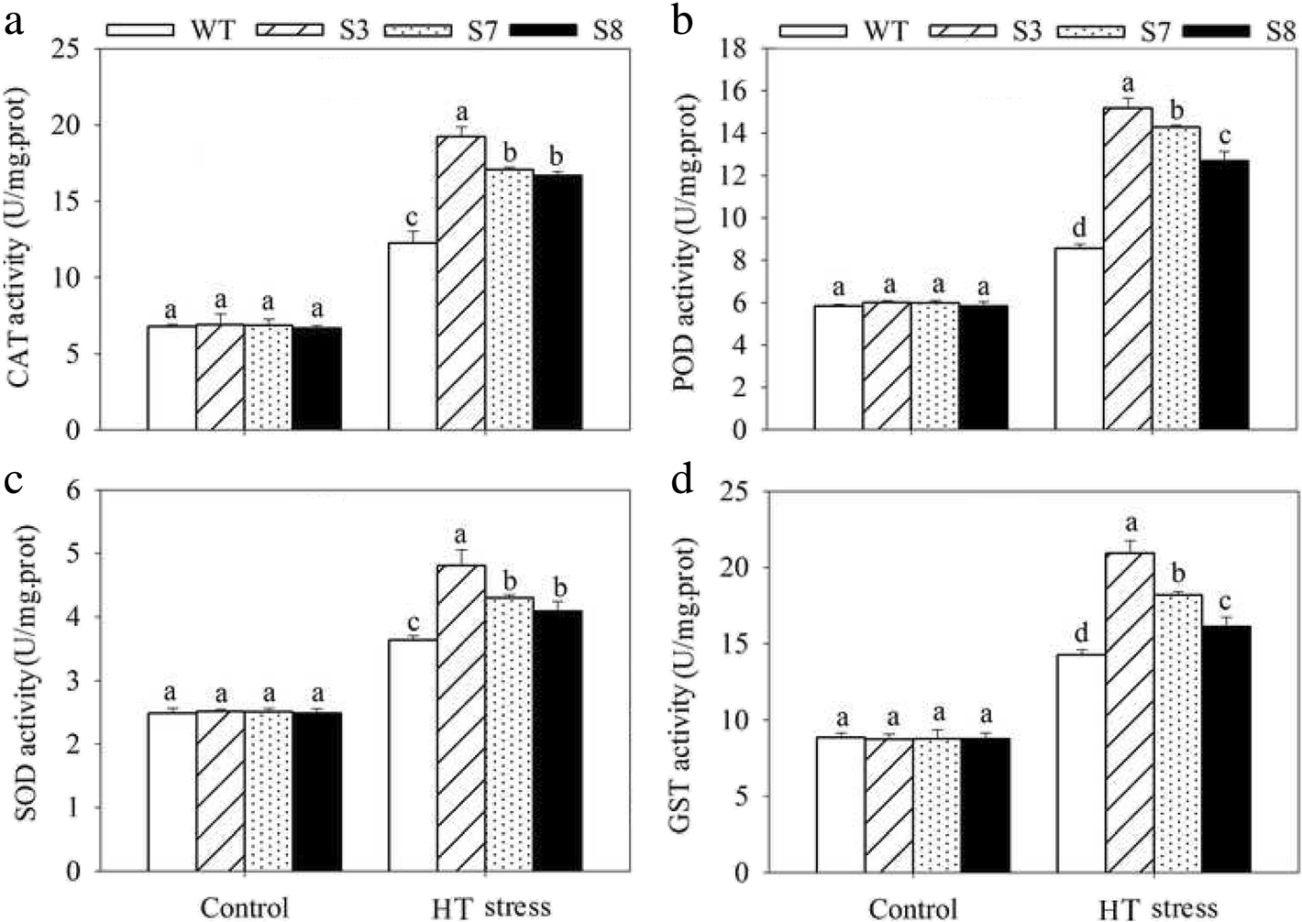
The antioxidases activities of five-week-old WT, S3, S7 and S8 exposed to HT stress for one-week. **a**, CAT activity; **b**, POD activity; **c**, SOD activity; **d**, GST activity. Significant differences (*p*<0.05) between WT and transgenic seedlings were indicated by lowercase

**Fig. 9:**
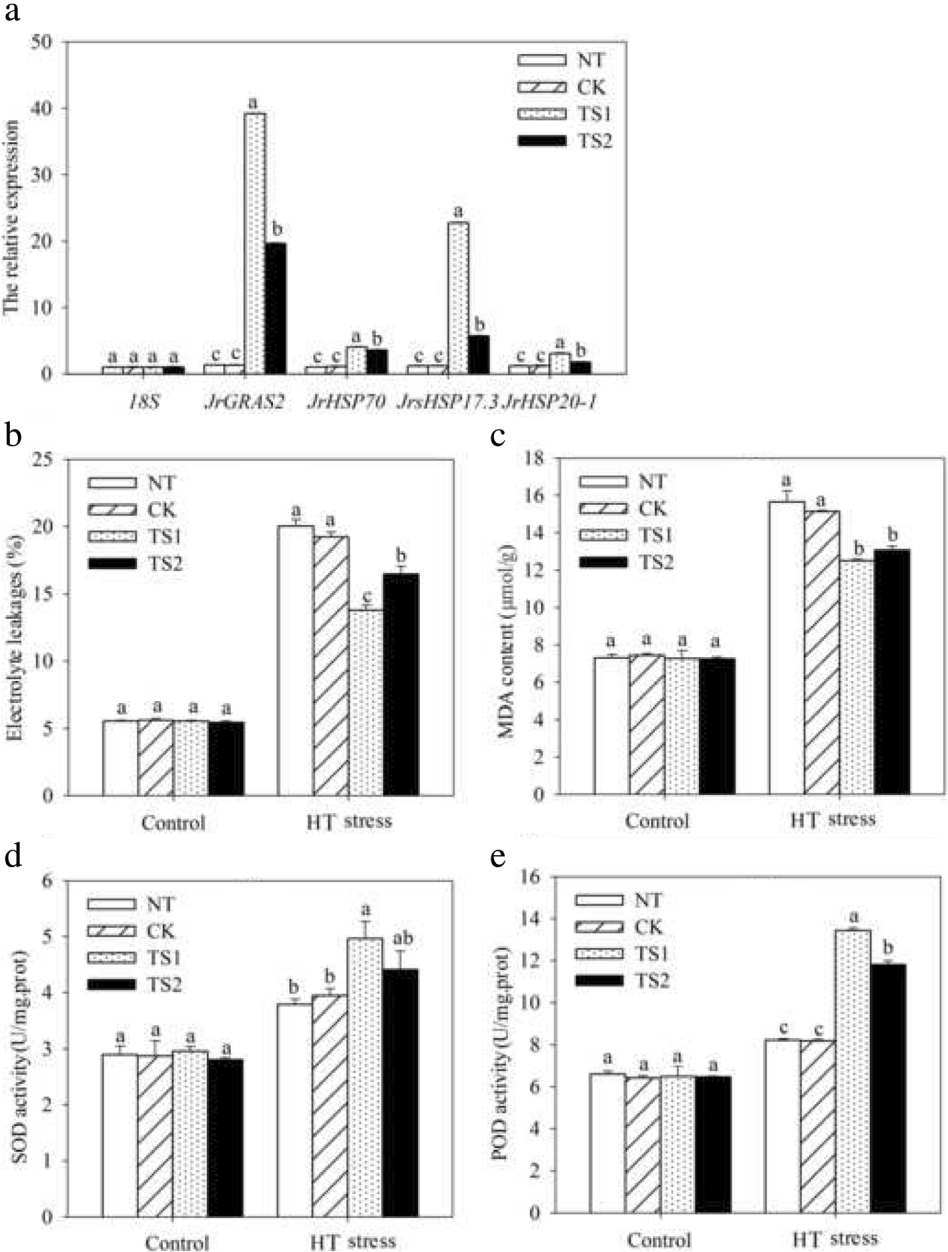
HT stress tolerance analysis by transient overexpression of *JrGRAS2* in walnut. NT, the leaves not transformed; CK, the leaves transformed by empty vector; TS1 and TS2 two lines transformed by *JrGRAS2*. **a**, the expression level of *JrGRAS2* and *HSP* genes in NT, CK, TS1 and TS2. **b**-**e**, the EL rate, MDA content, SOD and POD activities of NT, CK, TS1 and TS2 under normal and HT stress conditions. The significant differences (*p*<0.05) between transgenic lines and NT were indicated by lowercase

To complete characterize of the HT stress tolerance of *JrGRAS2*, it was overexpressed in *Arabidopsis*, and three lines with much higher expression level of *JrGRAS2* (S3, S7, and S8) (Additional file 3: Figure S2) were selected for analysis. Germination assays showed that the germination rates of S3, S7, and S8 were 1.14-~1.16-fold of WT exposed to HT treatment. The fresh weight of the germinated seedlings of the transgenic lines was average 1.34-fold of that of WT under HT stress (Fig. 6). The 5-week-old plants of WT, S3, S7, and S8 grow under normal conditions were exposed to 37°C for one-week, then the ROS accumulation was analyzed. Evans blue staining displayed deeper coloring on the leaves of WT, S3, S7, and S8 under HT conditions than under control conditions. What’s more, the staining of WT was deeper than three transgenic lines under HT stress (Fig. 7a). The electrolyte leakage (EL) rates, H_2_O_2_ and 3, 3'-Diaminobenzidine (DAB) content were all showed similar trend as the Evans blue staining (Fig. 7). The EL rate of WT was 2.11-, 1.99-, 1.76-fold of that of S3, S7, S8 under HT stress, accordingly; the corresponding H_2_O_2_ content of WT was 1.44-, 1.33-, 1.23-fold of that of S3, S7, S8; the MDA content of S3, S7, and S8 was only about 67.10% of that of WT. The activities of the antioxidase including catalase (CAT), peroxidase (POD), superoxide dismutase (SOD), and glutathione-s-transferase (GST) were also tested and showed a reverse tendency to MDA and H_2_O_2_ contents (Fig. 8). Under control conditions, WT and three transgenic lines were showed similar CAT, POD, SOD and GST activities, which were all lower compared to themselves under HT stress. However, the activities of CAT, POD, SOD and GST of S3, S7, and S8 were average 1.44-, 1.64-, 1.21- and 1.29-fold higher than those of WT, accordingly (Fig. 8). All these results demonstrated the positive role of *JrGRAS2* in plant HT stress tolerance. Moreover, transient expression method revealed that overexpression of *JrGRAS2* in walnut also decreased the MDA content and EL rate while increased the SOD and POD activities under HT stress compared to NT and CK lines (Fig. 9), further confirmed the positive role of *JrGRAS2* in walnut HT stress resistance.

### *JrGRAS2* improves plant HT tolerance involving in *HSP* expression

**Fig. 10:**
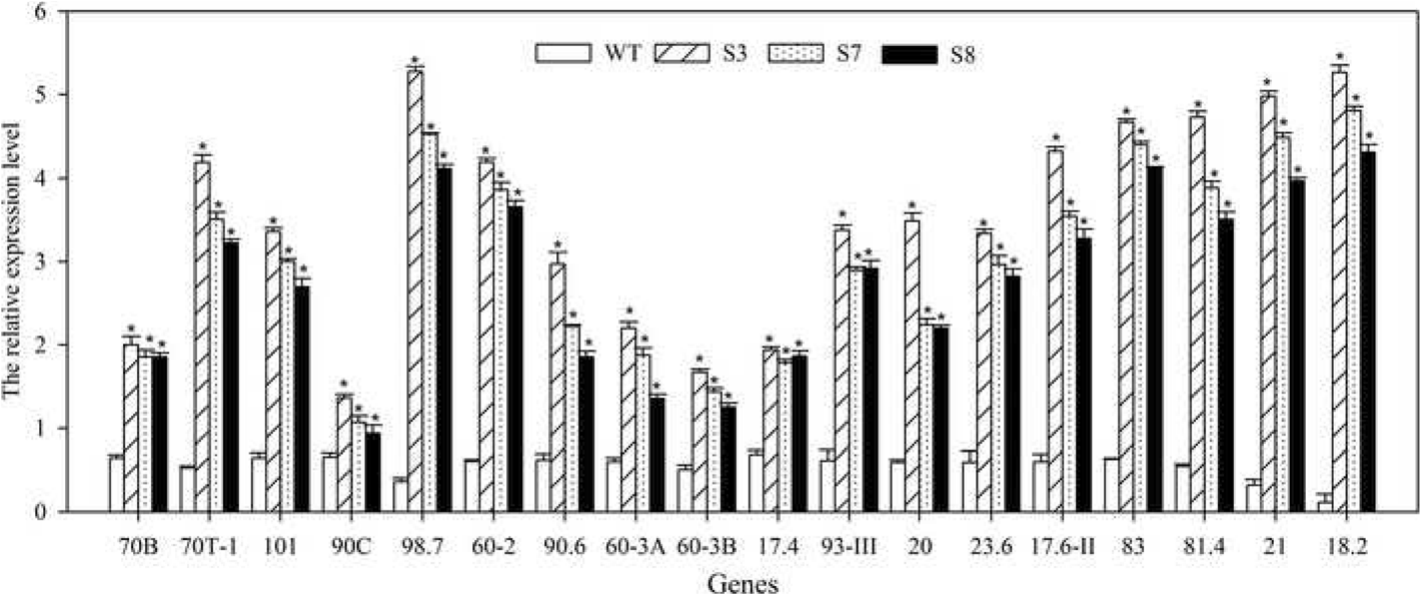
The expression of *HSP*s in *JrGRAS2* transgenic plants. The relative expression level is relative to the reference gene. Error bars were obtained from three replicates of qRT-PCR. The significant differences between WT and transgenic lines were marked as an asterisk (*p*<0.05). 70B, 70T-1, 101, 90C, 98.7, 60-2, 90.6, 60-3A, 60-3B, 17.4, 93-III, 20, 23.6, 17.6II, 83, 81.4, 21, and 18.2 presented the gene of *AtHSP70B*, *AtHSP70T-1*, *AtHSP101*, *AtHsp90C*, *AtHSP98.7*, *AtHSP60-2*, *AtHsp90.6*, *AtHSP60-3A*, *AtHSP60-3B*, *AtHSP17.4*, *AtHSP93-III*, *AtHSP20*, *AtHSP23.6*, *AtHSP17.6II*, *AtHSP83*, *AtHsp81.4*, *AtHSP21*, and *AtHSP18.2*, respectively

HSP family members are important HT stress response proteins. To understand whether the HT stress regulation of *JrGRAS2* relating to the expression of *HSP* genes, total 18 *HSP* genes from different subfamilies were chosen, and their transcription were determined in WT, S3, S7, and S8 using qRT-PCR method. The results showed that all the chosen *HSP*s (*AtHSP70B*, *AtHSP70T-1*, *AtHSP101*, *AtHsp90C*, *AtHSP98.7*, *AtHSP60-2*, *AtHsp90.6*, *AtHSP60-3A*, *AtHSP60-3B*, *AtHSP17.4*, *AtHSP93-III*, *AtHSP20*, *AtHSP23.6*, *AtHSP17.6II*, *AtHSP83*, *AtHsp81.4*, *AtHSP21*, *and AtHSP18.2*) were significantly up-regulated in *JrGRAS2* overexpression *Arabidopsis* plants, especial *AtHSP98.7*, *AtHSP18.2*, and *AtHSP21*, they were the top three induced genes (Fig. 10). Meanwhile, in transient overexpression lines TS1 and TS2, the expression of selected *HSP*s was enhanced (Fig. 9a), suggesting that HSP proteins participate in HT regulation of *JrGRAS2*.

## Discussion

Walnut is an important economic tree and affected by HT stress as other plant species, whose adaptation and response mechanisms on HT are currently insufficiently studied. Considering the abundant members of GRAS in walnut genome, the potential role of SCL subfamily GRAS TFs in HT stress response [34], and the HT stress response function and molecular mechanism of GRAS remains to be elucidated, in this study, a SCL GRAS was cloned from *J. regia* (named as *JrGRAS2*) and the up-stream regulation mechanism involving in HT stress response was characterized. The isolated up-stream promoter was heterologous transformed into *Arabidopsis* and homologous transient expressed in walnut, which displayed various GUS activities in different tissue parts and were enhanced by HT treatment (Fig. 1). Since the expression activity of the promoter is usually related to its function, for instance, the expression of *Tamarix hispida* V-ATPase c subunit (*ThVHAc1*) promoter was up-regulated by CdCl_2_ and *ThVHAc1* was further confirmed as Cd tolerance gene [42]; the promoter expression activity of Cd-resistance gene *JrVHAG1* (walnut V-ATPase G subunit) was induced by CdCl_2_ [43]; transformation of the banana aquaporin family gene *MaTIP1;2* promoter into *Arabidopsis* to assess its function indicated that it responds to both drought and salt stress treatments [44]; it can be learn that *JrGRAS2* is a potential HT stress tolerance gene.

The *JrGRAS2* promoter is consist of diverse *cis*-elements those were classified into different subfamilies. In detail, twenty-six, nineteen, thirteen, eight, seven and five different elements were belong to the classes of ‘ABA, Dehydration and salinity (osmotic) stress responsive’, ‘Tissue/organelles specific expression’, ‘Light responsive’, ‘Phytohormone responsive’, ‘Pathogen, elicitor and wound responsive’ and ‘Heat and cold stress related’, respectively (Additional file 2: Table S1), according to the reports from Vivekanand Tiwari et al [45], suggesting the potential abundant regulation function of this promoter. Among all the elements, DOFCOREZM motif is the most one (Additional file 2: Table S1). Meanwhile, Dof TFs were reported as the effective factors in plant stress response, such as salt, drought, cold and heat [46, 47], therefore, DOFCOREZM motif was picked up for yeast one-hybrid assay to screen for the potential up-stream regulators of *JrGRAS2*, and the results showed that JrDof3 could specially bind to the DOFCOREZM motif (Fig. 2) that functioned as an up-stream regulator of *JrGRAS2*, which were further confirmed by co-transient expression and Chip-PCR analysis (Figs. 3, 4).

The target gene responds to stress are usually regulated by the upstream regulators binding or unbinding to the motifs in the promoter [43, 45]. Since the promoter expression activity of *JrGRAS2* was enhanced by HT (Fig. 1) and *JrGRAS2* is speculated as a potential HT tolerance gene, the transcription of *JrDof3* and *JrGRAS2* exposed to HT treatment was analyzed by qRT-PCR and both showed positive response to HT stress (Fig. 5). For induced transcription is a prediction for potential function in plant stress response, such as: *Tamarix ThVHAc1* was up-regulated by CdCl_2_ then confirmed as a Cd tolerance gene [42]; walnut *JrGSTTau1* was induced by cold and further characterized as a chilling tolerance factor [48]; tomato *SlGRAS40* was upregulated by D-mannitol or NaCl, which was verified as drought and salt resistance TF [49], we can conclude that the HT stress response of *JrGRAS2* is controlled by *JrDof3* which may act as an up-stream regulator of *JrGRAS2* to either control or act together with *JrGRAS2* to participate in plant HT stress response.

To complete confirm the HT stress response function of *JrGRAS2*, it was overexpressed in *Arabidopsis* and three transgenic lines S3, S7 and S8 were selected for analysis. Interestingly, the expression of *JrGRAS2* is effective to improve plant HT stress tolerance which was demonstrated by germination ability, growth efficiency, ROS accumulation and antioxidant activity (Fig. 6, 7 and 8). And the parallel results were obtained in transient overexpression walnut lines exposed to HT treatment (Fig. 9). The similar results here were observed in the plants overexpression of *JrGSTTau1* that the transgenic plants accumulated less ROS, more biomass and higher activities of SOD, POD than WT plant under cold stress [50]; overexpression of the wheat F-Box protein gene *TaFBA1* enhanced heat stress tolerance in transgenic tobacco owing to the growth inhibition was reduced and photosynthesis was increased as compared with those in WT plants [51]; *Malus sieversii MsHsp16.9* is proved to be a protein chaperone that attenuate plant responses to severe stress via positively regulates antioxidant enzyme activity [52]. Therefore, we believe that *JrGRAS2* is a vital HT stress responsive gene for walnut tree in temperature adaption regulation.

Plant TFs usually participate in stress response by regulating downstream related genes [50], for instance, unconventional splicing of wheat *TabZIP60* could contribute to heat tolerance in transgenic plants by modulating the expression of ER stress-related genes [50]; *Vitis amurensis* GRAS TF *VaPAT1* confers abiotic stress tolerance via up-regulate stress-related genes such as *AtSIZ1*, *AtCBF1*, *AtATR1/MYB34*, *AtMYC2*, *AtCOR15A*, *AtRD29A* and *AtRD29B* [53]; *Tamarix* eukaryotic translation initiation factor 1A (*eIF1A*) connected to the expression of stress-related genes, *TOBLTP, GST*, *MnSOD*, *NtMPK9*, *poxN1* and *CDPK15*, in salt and drought stress response [54]. Since the HSP proteins were important HT stress-related members [6, 52, 55], the *Arabidopsis HSP* genes were identified from the TAIR database and analyzed in *JrGRAS2* overexpression *Arabidopsis* plants, whose transcription were enhanced in S3, S7 and S8 compared to those in WT plants (Fig. 10); meanwhile, the *HSP*s from walnut tree were also up-regulated by *JrGRAS2* in transient expression lines TS1 and TS2 (Fig. 9a); these findings were similar to the up-regulation of *HSP* genes in heat-tolerant csd1, csd2 and ccs plants while reduced in heat-sensitive transgenic plants expressing miR398-resistant forms of *CSD1*, *CSD2* or *CCS* [56]; overexpression of *HsfA1a* had positive effects on the tolerance to diverse stressors by promoting inducible of *Hsp* expression [57]. Therefore, based on these findings, we defined that *JrGRAS2* endows plant HT stress tolerance was partially by regulating the expression of *HSP* genes.

## Conclusion

The *JrGRAS2* promoter includes abundant stress related *cis*-elements and could be induced by HT stress, implying the positive role of *JrGRAS2* in HT stress response. Yeast one-hybrid, transient expression, Chip-PCR and qRT-PCR assays confirmed that JrDof3 was a potential up-stream regulator of *JrGRAS2* for HT stress resistance. Heterologous and homologous overexpression of *JrGRAS2* in *Arabidopsis* and walnut revealed that *JrGRAS2* is an effective TF for plant HT stress tolerance, which was involved in growth, ROS scavenging and antioxidant metabolism. These results indicated that *JrGRAS2* is an important candidate gene for plant HT stress tolerance in molecular breeding, and it will offer new insights to reveal the adverse stimulus adaptation mechanism of walnut trees.

## Methods

### Plant materials and treatments

Two-year-old grafted ‘Xiangling’ (a genotype of *J. regia* widely planted in China, in this study, the seedlings were obtained from Walnut Experimental Station, Northwest A & F University) seedlings were grown in a greenhouse with the relative humidity 70±5%, temperature 22±2°C, illumination cycle 14/10 h) [48], and treated with 37°C for 0 (control), 1, 3, 6, and 12 h. The leaves and roots were harvested independently and frozen in liquid nitrogen, then stored at -80°C for total DNA and RNA isolation, which were used as the template of promoter cloning and qRT-PCR analysis, accordingly. The treatment at each time point was applied three times and each treatment contained 9 seedlings.

### Identification and expression activity of the *JrGRAS2* promoter

The *J. regia* leaves was used to extract the genomic DNA by CTAB (cetyltrimethylammonium bromide) method with after ground in liquid nitrogen and cleaned in a 0.14 M NaCl solution. The DNA quality was confirmed by electrophoresing in 1.0 % agarose gel and staining with ethidium bromide (EB). The *JrGRAS2* promoter was identified from the walnut genome [41], and amplified by PCR reaction from the *J. regia* DNA. The PCR amplification parameters were set as follows: 30 s at 94°C followed by 35 cycles at 94°C for 30 s, 58°C for 30 s, 72°C for 90 s, and at 72°C for 7 min extending. The 20 μL PCR reaction mixture was generated according to manufacturer instructions (Takara Ex Taq®, Takara, Dalian, China). The *cis*-elements in the *JrGRAS2* promoter were categorized into different groups after the analysis using the online programs PLACE [58] and PLANTCARE [59]. The expression activity of *JrGRAS2* promoter (*JrGRAS2-P*) was functionally validated by a transgenic approach: The *35S* promoter was replaced by the *JrGRAS2* promoter to drive the expression of β-glucuronidase (*GUS*) gene in a pCAMBIA1301 vector to generate a recombinant construct pCAM-*JrGRAS2*-P, which was used for *Arabidopsis* plant transformation using *Agrobacterium*-mediated floral dip method [60]. Five-week-old transgenic seedlings were used to study the expression activity and level through GUS activity determination and staining [42, 61] under normal and HT stress (37°C). Meanwhile, the pCAM-*JrGRAS2*-P was transient transformed into walnut leaves using *Agrobacterium*-mediated method. The EHA105(pCAM-*JrGRAS2*-P) cells were grown to OD_600_=0.8~1.0, then diluted to OD_600_=0.05~0.1 with 1/2MS (Murashige and Skoog) liquid medium plus with 100 μM acetosyringone (AS). The three-month-old *J. regia* leaves were immersed in this solution and incubated for 8~10 h at 25°C with 40~50 rpm shaking, rinsed third with 1/2MS and incubated in fresh 1/2MS plus with 100 μM AS for 52~55 h. Fresh 1/2MS plus with 100 μM AS was added immediately to keep the OD_600_<1.0. Then the GUS activities of the transformed leaves treated by 25 and 37°C were determined. Every treatment was replicated three times and each replicate contained at least 15 seedlings.

### Characterization of the potential upstream regulator of *JrGRAS2*

The core sequence of DOFCOREZM motif is "AAAG", and total 26 DOFCOREZM elements were found in the *JrGRAS2* promoter (Additional file 2: Table S1, Additional file 1: Figure S1). Yeast one-hybrid assays were employed to identify the up-stream TFs capable of recognizing the DOFCOREZM motif. Three tandem copies of "AAAG" were cloned into pHis2 vector (pHis2-DOF) (Fig. 2a) [43]. The Dof TFs were identified from the *J. regia* transcriptome and then cloned into the pGADT7-Rec2 vector to generate a cDNA library for use in yeast one-hybrid assays [43]. The interactions between the DOFCOREZM motif and positive clones were further confirmed by examining the binding of mutated motif or promoter segments to the potential Dof TFs. I.e, the DOFCOREZM "AAAG" was mutated to "CCCA" and inserted into pHis2 (pHis2-DOF-M); *JrGRAS2* promoter fragments including the DOFCOREZM motif (pHis2-DOF-S), containing the mutated DOFCOREZM motif (pHis2-DOF-M1), and excluding the DOFCOREZM motif (pHis2-DOF-M2) were all independently cloned into pHis2 [43]; Then the interactions between pHis2-DOF-M, or pHis2-DOF-S, or pHis2-DOF-M1, or pHis2-DOF-M2 and candidate positive clones were verified on the TDO plates with 50 mM 3-AT. The construct of p53His2 was set as a control in the yeast one-hybrid assays [42, 61].

Furthermore, above interactions were examined in tobacco seedlings by transient co-expression method [42, 43]. The DOFCOREZM motif (DOF), DOF-M, DOF-S, DOF-M1, DOF-M2 were each fused with a *CaMV35S*-46 minimal promoter to construct recombinants, which were further independently cloned into the pCAMBIA1301 vector to drive the *GUS* gene expression (reporters) (Fig. 3a) [43]. The ORF of the screened TF*-- JrDof3* was cloned into pROKII vector under the control of a *35S* promoter (pROKII-*JrDof3*) to generate the effecter (Fig. 3a) [43], which was transiently co-transformed with each of the reporters in tobacco leaves using *Agrobacterium*-mediated transformation method, then GUS activities of the co-transformed tobacco leaves were measured to evaluate the interactions [61, 62]. Every co-transformation was replicated three times and each replicate contained at least 20 leaves. Meanwhile, the direct binding of JrDof3 to the *JrGRAS2* promoter was analyzed by Chip-PCR using the *JrGRAS2* promoter segment containing the DOFCOREZM motif (S), or containing the mutated DOFCOREZM motif (M1), or excluding the DOFCOREZM motif (M2). The Chip assay was performed according to instructions of ChIP Assay Kit (Beyotime Biotechnology, Shanghai, China) (http://www.beyotime.com) and the published method [63]. All the used primers are listed in Additional file 4: Table S2.

### RNA isolation and HT stress response analysis of *JrGRAS2* and *JrDof3*

Total RNA of each sample was isolated using the CTAB method and reverse-transcribed into cDNA [64], which was used as the template of qRT-PCR after diluting to 1/10 of the original concentration with sterile water. The *18S rRNA* was used as a reference gene [65]. The qRT-PCR was performed in a CFX96 Touch^TM^ Real-Time PCR Detection System (Bio-Rad Laboratories, Redmond, WA) [48]. The 20 μL reaction mixture contained 10 μL of SYBR Green Real-time PCR Master Mix (CWBIO), 0.5 μM of each forward and reverse primer (Additional file 4: Table S2), 2 μL cDNA template (equivalent to 100 ng of total RNA). The amplification was applied using the following cycling parameters: one cycle of 94°C for 30 s, followed with 44 cycles at 94°C for 12 s, 60°C for 30 s, 72°C for 40 s, and at 81°C for 1 s plate reading. Three independent experiments were applied to ensure the reproducibility of qRT-PCR results. The relative expression levels were calculated based on the threshold cycle according to the 2^-ΔΔ*CT*^ method [66].

### HT stress tolerance analysis of *JrGRAS2* in transgenic *Arabidopsis* plants

The open reading frame (ORF) of *JrGRAS2* was amplified using the primers of *JrGRAS2*-F/-R (Additional file 4: Table S2), which was further confirmed by sequencing. The *JrGRAS2* amplified ORF fragment was digested by *Xba*I and *Kpn*I and then cloned into pROKII vector under the control of a CaMV35S promoter to construct 35S::*JrGRAS2*. The EHA105 harboring recombinant 35S::*JrGRAS2* was cultivated and used for transformation of *Arabidopsis* plants. Kanamycin-resistant transformed seedlings were detected by PCR, whose expression level was analyzed by qRT-PCR. Three transgenic lines with highest expression of *JrGRAS2* (Line S3, S7, and S8) were chosen for further analysis.

Firstly, the seed germination ability and growth performance exposed to HT were analyzed. The seeds of WT, S3, S7, and S8 were sown on 1/2MS agar medium under 24°C or 37°C for 12 d. Then the germination rate and fresh weight were recorded, respectively. Next, the ROS accumulation was assessed. The 5-week-old plants of WT, S3, S7, and S8 grow under normal conditions were transferred to 37°C for another one-week, then the leaves were stained by Evans blue, and the corresponding EL rate, H_2_O_2_ and MDA contents were tested. Lastly, the activities of the antioxidases including CAT, POD, SOD, and GST were determined under the treatments as ROS accumulation assessment.

### Transient expression analysis of *JrGRAS2* to HT stress

To further confirm the HT stress response ability of *JrGRAS2*, the recombinant 35S::*JrGRAS2* was transient transformed into walnut. The EHA105 (35S::*JrGRAS2*) and EHA105 (pROKII) (CK) cells were grown to OD_600_=0.8, then diluted to OD_600_=0.05 in 1/2MS liquid medium supplementing with 100 μM AS. Then the three-mouth-old *J. regia* leaves were transformed as the method using for pCAM-*JrGRAS2*-P transient transformation [67]. The non-transformed (NT) and CK lines were used as control. The expression level of *JrGRAS2* was checked to confirm the transformation. The EL rate, MDA content, SOD activity and POD activity were tested. The transcription of walnut *HSP* genes (*JrHSP70*, *JrsHSP17.3*, *JrHSP20-1*) were analyzed to understand the regulation of *JrGRAS2* whether relating to *HSP*s. All experiments were performed three times, each replicate contained 15 seedlings.

### Transcription analysis of the *HSP*s regulating by *JrGRAS2*

The HSP proteins were identified from the *Arabidopsis* genome online database --TAIR (The *Arabidopsis* Information Resource, http://www.arabidopsis.org/). Total 18 *HSP* genes from different subfamilies were selected, they are *AtHSP70B*, *AtHSP70T-1*, *AtHSP101*, *AtHsp90C*, *AtHSP98.7*, *AtHSP60-2*, *AtHsp90.6*, *AtHSP60-3A*, *AtHSP60-3B*, *AtHSP17.4*, *AtHSP93-III*, *AtHSP20*, *AtHSP23.6*, *AtHSP17.6II*, *AtHSP83*, *AtHsp81.4*, *AtHSP21*, and *AtHSP18.2*. Their expression levels were confirmed in *JrGRAS2* transgenic *Arabidopsis* plants using qRT-PCR. *Actin2* was used as an internal control. All the primers and gene accession number were included in Additional file 5: Table S3.

### Statistical analysis

All experiments were repeated three times, all of the data were analyzed using the Statistical Package for Social Science (SPSS) (SPSS, Chicago, Illinois), the sample variability is reported as standard deviation (S.D.). The differences between the transgenic and WT lines were evaluated using Tukey’s multiple comparison test with the significance level set at *p*<0.05.

## Additional files

Additional file 1: **Figure S1.** The *JrGRAS2* promoter sequence and main *cis*-elements existing in the promoter that predicted by PLACE and PLANTCARE. (JPG 2941 kb)

Additional file 2: **Table S1.** All the motifs existed in the promoter that predicted and classified according to the online programs of PLACE and PLANTCARE. (PDF 89 kb)

Additional file 3: **Figure S2.** The expression level of *JrGRAS2* in transgenic *Arabidopsis*. 1-12, twelves transgenic lines. (JPG 126 kb)

Additional file 4: **Table S2.** The primers used for pHIS2, pGADT7-Rec2, pCAMBIA1301, and pROKII recombinant vector construction, and qRT-PCR analysis of *JrGRAS2*, *JrDof3* as well as *18S rRNA* gene. (PDF 138 kb)

Additional file 5: **Table S3.** The primers used for qRT-PCR analysis of *HSP* genes. (PDF 133 kb)

## Abbreviations

ABA: Abscisic acid
CAT: Catalase
CTAB: Cetyltrimethylammonium ammonium bromide
EL: Electrolyte leakage
GST: Glutathione-S-transferase
HSP: Heat shock protein
HT: High temperature
MDA: Malondialdehyde
MS: Murashige and skoog
POD: Peroxidase
qRT-PCR: Quantitative real-time PCR
ROS: Reactive oxygen species
S.D.: Standard deviation
SD: Synthetic drop-out medium
SOD: Superoxide dismutase
TF: Transcription factor
3-AT: 3-amino-1, 2, 4-triazole
