# Supplementary figures and images for: The walnut transcription factor JrGRAS2 contributes to high temperature stress tolerance involving in Dof transcriptional regulation and HSP protein expression
Source: BMC Plant Biol. 2018 Dec 20;18:367. doi: 10.1186/s12870-018-1568-y (PMC6302389; doi:10.1186/s12870-018-1568-y)

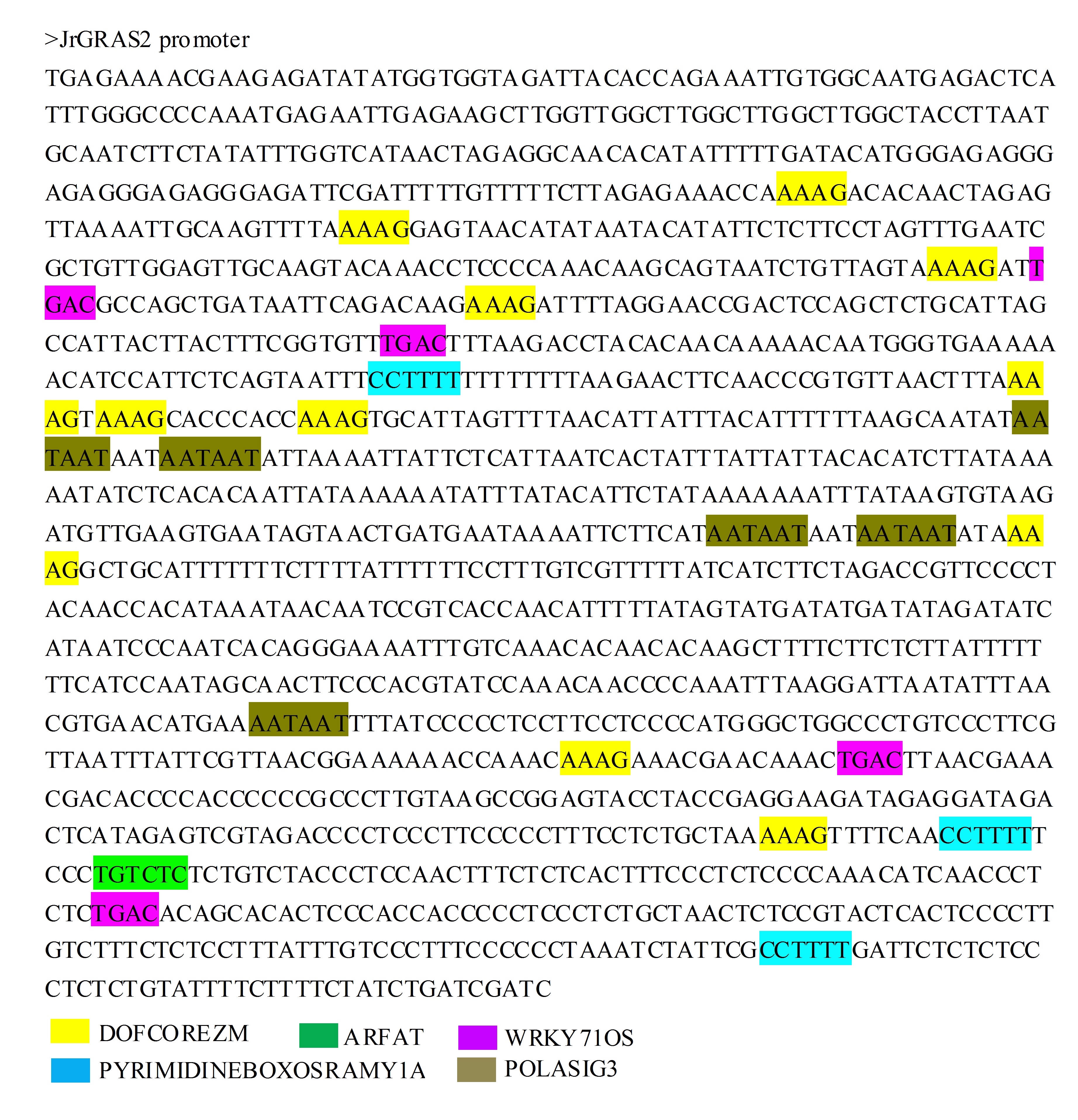

Supplement: Supplementary file 1 — Figure S1. The JrGRAS2 promoter sequence and main cis-elements existing in the promoter that predicted by PLACE and PLANTCARE. (JPG 2941 kb) [file 12870_2018_1568_MOESM1_ESM.jpg]

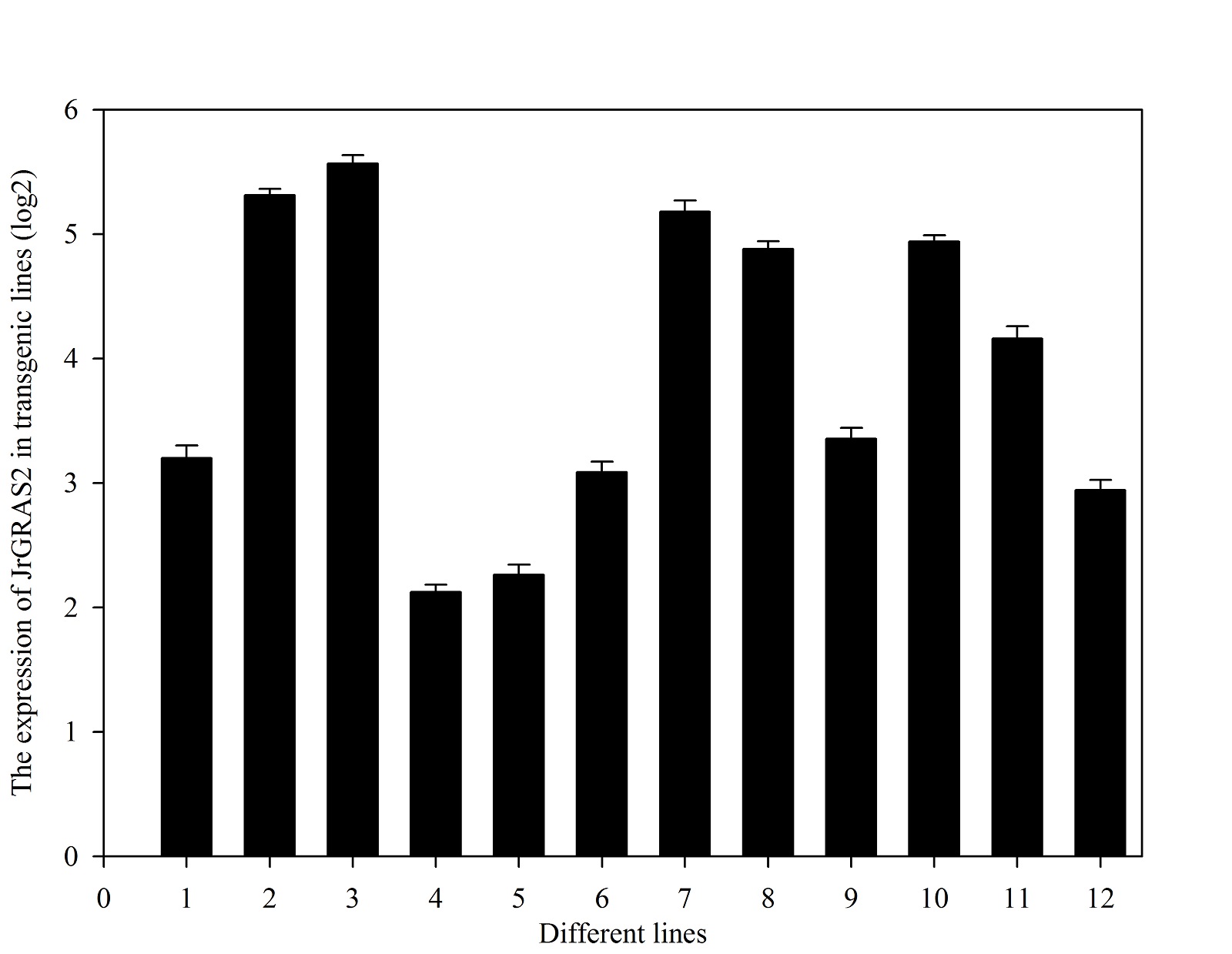

Supplement: Supplementary file 3 — Figure S2. The expression level of JrGRAS2 in transgenic Arabidopsis. 1-12, twelves transgenic lines. (JPG 126 kb) [file 12870_2018_1568_MOESM3_ESM.jpg]
