# Supplementary material for: The walnut transcription factor JrGRAS2 contributes to high temperature stress tolerance involving in Dof transcriptional regulation and HSP protein expression
Source: BMC Plant Biol. 2018 Dec 20;18:367. doi: 10.1186/s12870-018-1568-y (PMC6302389; doi:10.1186/s12870-018-1568-y)
Supplement: Supplementary file 2 — Table S1. All the motifs existed in the promoter that predicted and classified according to the online programs of PLACE and PLANTCARE. (PDF 89 kb) [file 12870_2018_1568_MOESM2_ESM.pdf]

TableS1 All the motifs existed in the promoter that predicted and classified according to the online programs of PLACE and PLANTCARE.

Category	Motif	Position	Consensus Sequence	Function
ABA, Dehydration & salinity (osmotic) stress responsive	ABRELATERD1	997(-), 1037(+)	ACGTG	ABRE-like sequence; ABA and dark induced senescence
	ACGTATERD1	998(-), 998(+), 1037(-), 1037(+)	ACGT	Etiolation induced expression of erd1
	CURECORECR	1192(-), 1192(+), 1391(-), 1391(+), 320(-), 320(+)	GTAC	Oxygen deficiency responsive gene expression through copper-sensing signal transduction pathway
	DPBFCOREDCDC3	530 (-),951 (+), 33 (+)	ACACNNG	ABA inducible bZIP transcription factor DPBF-1 & 2 binding site
	GT1GMSCAM4	209(-), 479(+), 801(-), 813(-), 974(-), 1118(+), 1277(-)	GAAAAA	Salt and pathogen inducible GT-1 motif found in soybean
	MYB1AT	221(+), 1122(+)	WAACCA	MYB recognition site found in the promoters of the dehydration-responsive gene
	MYB2CONSENSUSAT	747(+), 1113(+)	YAACKG	MYB recognition site found in the promoter of dehydration responsive gene rd22
	MYBCOREATCYCB1	845(-), 1114 (+)	AACGG	Myb core in the 18 bp sequence which is able to activate reporter gene without leading to M-phase-specific expression
	MYBCORE	305(+), 348(+), 747(-), 1113(-)	CNGTTR	Binding site MYB that is responsive to water stress or involved in regulation of flavonoid biosynthesis
	MYBST1	1213(+), 1001(-), 1058(-)	GGATA	Core motif of MybSt1 (a potato MYB homolog) binding site
	MYBPLANT	1123(+)	MACCWAMC	Plant MYB binding site; Consensus sequence related to box P in promoters of phenylpropanoid biosynthetic genes
	MYBPZM	91(-), 1195(+)	CCWACC	Core of consensus maize P (myb homolog) binding site
	MYB2AT	747(+)	TAACTG	Binding site for ATMYB2, an Arabidopsis MYB homolog
	BOXLCOREDCPAL	1194(+)	ACCWWCC	Consensus of the putative "core" sequences of box-L-like sequences in carrot (D.c.) PAL1 promoter region; DCMYB1 bound to these sequences in vitro
	EECCRCAH1	311(+)	GANTTNC	Consensus motif of the two enhancer elements, EE-1 and EE-2, Binding site of Myb transcription factor LCR1
	MYCCONSENSUSAT	369(-), 369(+), 59(-), 59(+), 70(-), 70(+)	CANNTG	MYC recognition site found in promoter of dehydration responsive genes
	PREATPRODH	56(+), 1219(+)	ACTCAT	"PRE (Pro- or hypoosmolarity-responsive element) found in the promoter region of proline dehydrogenase (ProDH) gene
	DRE2COREZMRAB17	402(+)	ACCGAC	DRE2 core found in maize rab17 gene promoter; rab17 is expressed during late embryogenesis, and is induced by ABA
	DRECRTCOREAT	402(+)	RCCGAC	ore motif of DRE/CRT cis-acting element found in many genes
	SITEIIATCYTC	1079(+), 63(+)	TGGGCY	found in the promoter regions of cytochrome genes (Cytc-1, Cytc-2), related to oxidative phosphorylation
Conserved promoter motifs	CAATBOX1	79(-), 123(+), 248(-), 361(-), 471(+), 600(+), 681(+), 871(+), 923(+), 98 (+), 42(-), 49(+)	CAAT	CAAT promoter consensus sequence
	INRNTPSADB	1313(+), 1394(+), 77(-), 736(-)	YTCANTYY	Inr motif responsible for TATA independent initiation of transcription
	TATABOX5	809(+), 865(-), 969(+), 1049(-), 1419(+), 582(+)	TTATTT	TATA box found in the 5'upstream region of pea (Pisum sativum) glutamine synthetase gene
	TATABOXOSPAL	103 (+)	TATTTAA	Binding site for OsTBP2

	TATABOX2	695(-), 716(-)	TATAAAT	found in the 5' upstream region of pea legA gene; critical for accurate initiation
	TATABOX3	1026(-)	TATTAAT	"TATA box"; TATA box found in the 5'upstream region of sweet potato sporamin A gene;
Light responsive	EBOXBNNAPA	59(-), 59(+), 70(-), 70(+), 369(-), 369(+)	CANNTG	E-Box drive light responsive expression and control tissue-specific activation
	GATABOX	15(+), 167(+), 374(+), 672(-), 831(-), 899(+), 904(+), 910(+), 912(-), 1001(-), 1058(-), 1207(+), 1214(+), 1488(-)	GATA	Light responsive expression (found in promoter of all LHCII type I Cab genes
	GT1CONSENSUS	209(-), 374(+), 479(+), 501(-), 801(-), 813(-), 814(-), 829(-), 932(+), 933(+), 974(-), 1047(+), 1056(-), 1117(+), 1118(+), 1277(-), 1278(-), 1477(-)	GRWAAW	GT-1 motif; Light regulated expression
	IBOXCORE	37 (+), 830(-), 1057(-)	GATAA	Conserved sequence upstream to the light regulated genes
	PALBOXPPC	135 (+)	YTYYMMCM AMCMMC	Box P; conferred elicitor or light responsiveness
	SORLIP5AT	1394(-)	GAGTGAG	one of "Sequences Over-Represented in Light-Induced Promoters (SORLIPs) in Arabidopsis
	SORLIP2AT	64(+), 65(-), 1085(-)	GGGCC	Sequences over-represented in light- induced promoters
	SORLIP1AT	45(-)	GCCAC	Computationally identified phyA-induced motifs
	BOXCPSAS1	1359(+)	CTCCCAC	Box C in pea asparagine synthetase (AS1) gene; AS1 is negatively regulated by light
	-10PEHVPSBD	282(+), 629 (+)	TATTCT	Found in chloroplast psbD gene promoter, which encodes a chlorophyll-binding protein that is activated by blue, white or UV-A light
	REALPHALGLHCB21	1123(+), 89(-), 222(+)	AACCAA	Required for phytochrome regulation
	TBOXATGAPB	561(-)	ACTTTG	Found in the GAPB gene promoter; Mutations in the "Tbox" resulted in reductions of light-activated gene transcription
	MARTBOX	780(-), 807(+), 969(+), 507(+), 508(+), 509(+)	TTWTWTTWT T	T-Box; Motif found in SAR (scaffold attachment region) or MAR (matrix attachment region)
Phytohormone responsive	ARR1AT	28(+), 124(-), 195(+), 200(+), 300(-), 345(-), 359(+), 391(+), 639(-), 872(-), 918(-), 924(-), 1024(+), 1441(-), 1456(+)	NGATT	Cytokinin regulated ARR1 binding site
	ARFAT	1285(+)	TGTCTC	ARF (auxin response factor) binding site found in the promoters of primary/early auxin response genes of Arabidopsis thaliana
	PYRIMIDINEBOXOSR AMY1A	260(-), 505(+), 788(-), 1275(+), 1451(+)	CCTTTT	Pyrimidine box found in rice (O.s.) alpha-amylase (RAmy1A) gene; Gibberellin-respons cis-element of GARE and pyrimidine box are partially involved in sugar repression
	PYRIMIDINEBOXHVEP B1	812(+), 1117(-)	TTTTTTCC	"Pyrimidine box" found in the barley (H.v.) EPB-1 (cysteine proteinase) gene promoter; Located between -120 to -113; Required for GA induction
	GARE1OSREP1	347(-)	TAACAGA	"Gibberellin-responsive element (GARE)" found in the promoter region of a cystein proteinase (REP-1) gene in rice
	T/GBOXATPIN2	1036(+)	AACGTG	"T/G-box" found in tomato proteinase inhibitor II (pin2) and leucine aminopeptidase (LAP) genes; Involved in jasmonate (JA) duction of

				these genes
	CAREOSREP1	311(-)	CAACTC	Gibberellin regulated proteinase expression
	TATCCAOSAMY	1001(+)	TATCCA	Found in alpha-amylase promoters of rice; Binding sites of MYBs which mediate sugar and hormone regulation of alpha-amylase gene expression
Pollen and embryos specific	POLLEN1LELAT52	1133(+), 1308(-), 1409(-), 1479(-), 1484(-)	AGAAA	Pollen specific activation
	GTGANTG10	477(+), 641(-), 676(-), 739(+), 878(-), 926(-), 1039(+), 1314(-), 1395(-)	GTGA	GTGA motif in late pollen gene g10 promoter
	POLLEN1LELAT52	3(+), 38(+), 211(-), 219(+), 387(+), 803(-), 960(-)	AGAAA	One of two co-dependent regulatory elements responsible for pollen specific activation of tomato
Miscellaneous	DOFCOREZM	227(+), 261(+), 357(+), 389(+), 434(-), 448(-), 506(-), 540(-), 545(+), 550(+), 562(+), 789(+), 806(-), 819(-), 958(-), 1131(+), 1250(-), 1264(+), 1276(-), 1452(-), 1482(-), 1307(-), 1317(-), 1408(-), 1417(-), 1429(-)	AAAG	Core sequence of DOF transcription factor binding site
	RAV1AAT	462(+), 732(-), 882(+), 949(+), 153(+), 306(-)	CAACA	RAV1 transcription factor binding site
	SURECOREATSULTR1 1	53(+), 1286(-)	GAGAC	Core of sulphur responsive element
	SEF1MOTIF	693 (+)	ATATTTAWW	Found in 5'-upstream region of soybean beta-conglicinin (7S globulin) gene
	SEF4MOTIFGM7S	826(+), 886(+), 161(+), 202(+), 465(-), 666(-)	RTTTTTR	Found in 5'upstream region of 7S globulin gene; "Binding with SEF4
	POLASIG3	771(+), 774(+), 777(+), 780(+), 1050(+), 581(-), 606(+), 609(+), 612(+), 615(+), 627(-), 650(-)	AATAAT	Plant polyA signal; Consensus sequence for plant polyadenylation signal
	POLASIG1	756(+), 808(-), 1104(-), 1418(-), 647(-)	AATAAA	PolyA signal; found in legA gene of pea, rice alpha-amylase
Tissue/organelles specific expression	BOXIINTPATPB	1485(-), 128(-), 704(-)	ATAGAA	Box II motifs on some non-consensus type plastid promoters
	CACTFTPPCA1	745(-), 894(-), 1191(-), 1315(+), 1357(+), 1392(+), 1396(+), 266(-), 319(-), 342(-), 353(-), 428(+), 432(+), 497(-), 547(-), 564(-), 642(+), 723(-), 738(-)	YACT	Mesophyll specific expression in C4 plants
	NODCON2GM	966(+), 11(-), 286(+)	CTCTT	Nodule specific expression
	NODCON1GM	357(+), 389(+)	AAAGAT	One of two putative nodulin consensus sequences
	OSE1ROOTNODULE	389(+), 357(+)	AAAGAT	One of the consensus sequence motifs of organ-specific elements (OSE) characteristic of the promoters activated in infected cells of root nodules
	OSE2ROOTNODULE	966(+), 11(-), 286(+)	CTCTT	Nodule and organ specific expression after infection
	SP8BFIBSP8BIB	742(-)	TACTATT	One of SPBF binding site (SP8b); found in the 5' upstream region genes coding for sporamin and beta-amylase
	RHERPATEXPA7	1037(-)	KCACGW	"Right part of RHEs (Root Hair-specific cis-Elements)" conserved among the Arabidopsis thaliana A7 (AtEXPA7) orthologous
	PROXBBNNAPA	439(-)	CAAACACC	"prox B found in napA gene of Brassica napus (B.n.); Required for seed specific expression and ABA responsiveness;
	NAPINMOTIFBN	655(+)	TACACAT	Sequence found in 5' upstream region (-6, -95, -188) of napin (2S albumin) gene in Brassica napus (B.n.); Interact with a protein present in crude nuclear extracts from developing B. napus seeds;
	AMYBOX1	347(-)	TAACARA	"amylase box"; Conserved sequence found in 5'-upstream region of

				alpha-amylase gene of rice, wheat, barley;
	AACACOREOSGLUB1	1140(+)	AACAAAC	Core of AACA motifs found in rice (O.s.) glutelin genes, involved in controlling the endosperm-specific expression;
	CANBNNAPA	943(+), 440(-)	CNAACAC	Core of "(CA)n element" in storage protein genes in Brasica napus (B.n.); embryo- and endosperm-specific transcription of napin (storage protein) gene, napA; seed specificity;
	2SSEEDPROTBANAPA	440(-), 943(+)	CAAACAC	Conserved in many storage-protein gene promoters; May be important for high activity of the napA promoter;
	XYLAT	1129(+)	ACAAAGAA	cis-element identified among the promoters of the "core xylem gene set"
	NTBBF1ARROLB	447(+), 539(+)	ACTTTA	NtBBF1(Dof protein from tobacco) binding site in Agrobacterium rhizogenes (A.r.) rolB gene; Required for tissue-specific expression and auxin induction;
	SREATMSD	1057(+)	TTATCC	"sugar-repressive element (SRE)" found in 272 of the 1592 down-regulated genes after main stem decapitation in Arabidopsis;
	ROOTMOTIFTAPOX1	783(-), 1029(-), 1030(+), 132(+), 159(+), 281(+), 601(-), 618(-), 619(+), 670(-), 692(-), 693(+)	ATATT	Root specific expression
	TAAAGSTKST1	1417(-), 448(-), 540(-), 549(+)	TAAAG	Guard cell specific expression mediated by Dof1 protein
Pathogen, elicitor and wound responsive	WBOXATNPR1	941(-), 362(+), 444(+)	TTGAC	WB Box
	WBOXNTERF3	445(+), 1147(+), 138(-)	TGACY	W box found in the promoter region of ERF3 gene; May be involved in activation of ERF3 gene by wounding
	WRKY71OS	363(+), 445(+), 877(-), 941(-), 1147(+), 1347(+), 139(-)	TGAC	A core of TGAC-containing W-box; Involved in the GA signalling pathway; Bind specifically to TGAC-containing W box within the PR10 genes
	WBOXHVISO1	445(+), 1147(+)	TGACT	SUSIBA2 bind to W-box element in iso1 (encoding isoamylase1) promoter;
	WBBOXPCWRKY1	443(+)	TTTGACY	"WB box"; WRKY proteins bind specifically to the DNA sequence motif (T)(T)TGAC(C/T), which is known as the W box
	SEBFCONSSTPR10A	1284(+)	YTGTCWC	Binding site of the potato silencing element binding factor (SEBF) gene found in promoter of pathogenesis-related gene (PR-10a);
	WBOXNTCHN4	1146(+)	CTGACY	"W box" identified in the region between -125 and -69 of a tobacco class I basic chitinase gene CHN48
Heat and cold stress realted	UPRMOTIFIIAT	982(+)	CCNNNNNNNNNNNNNCCACG	"Motif II" in the conserved UPR (unfolded protein response) cis-acting element in Arabidopsis genes coding for SAR1B, HSP-90, SBR-like, Ca-ATPase 4, CNX1, PDI, etc.; See S000425,
	CBFHV	402(+)	RYCGAC	Cold responsive DRE (Binding site of HvCBF1)
	CCAATBOX1	922 (+),982 (+)	CCAAT	CAAT box found in the promoter elements of heat shock proteins
	LTRECOREATCOR15	403(+)	CCGAC	Core of low temperature responsive element (LTRE) of cor15a gene; ABA responsiveness
	LTRE1HVBLT49	435(-)	CCGAAA	"LTRE-1" (low-temperature-responsive element) in barley (H.v.) blt4.9 gene promoter
Other stress response	ANAERO1CONSENSUS	205(-), 1127(+)	AAACAAA	One of 16 motifs found in silico in promoters of 13 anaerobic genes involved in the fermentative pathway (anaerobic set 1)(Mohanty et al., 2005)

Other function	UP1ATMSD	60(-)	GGCCCAWWW	"Up1" motif found in 162 of the 1184 up-regulated genes after main stem decapitation in Arabidopsis; W=A/T
	CARGCW8GAT	158(-), 158(+), 1015(-), 1015(+)	CWWWWWWWWG	A variant of CArG motif, with a longer A/T-rich core; Binding site for AGL15 (AGAMOUS-like 15); W=A/T;
	CEREGLUBOX2PSLEGA	1266(-)	TGAAAACT	"cereal glutenin box" in pea legumin gene (legA); sequence homologous to the cereal glutenin gene control element ("-300 element")
	BIHD1OS	940(+), 1347(-)	TGTCA	Binding site of OsBIHD1, a rice BELL homeodomain transcription factor;
	CARGNCAT	1014(-), 1014(+)	CCWWWWWWWWGG	Noncanonical CArG motif (CC-Wx8-GG) found in the promoter region of DTA1 (AtGA2ox6)
	EVENINGAT	668(+)	AAAATATCT	"Evening element" found 46 times in the promoters of 31 cycling genes in Arabidopsis thaliana; Required for circadian control
	ASF1MOTIFCAMV	363(+), 876 (-)	TGACG	"ASF-1 binding site" in CaMV 35S promoter; ASF-1 binds to two TGACG motifs
	E2FCONSENSUS	1430(+)	WTTSSCSS	"E2F consensus sequence" of all different E2F-DP-binding motifs that were experimentally verified in plants
