# Supplementary material for: The walnut transcription factor JrGRAS2 contributes to high temperature stress tolerance involving in Dof transcriptional regulation and HSP protein expression
Source: BMC Plant Biol. 2018 Dec 20;18:367. doi: 10.1186/s12870-018-1568-y (PMC6302389; doi:10.1186/s12870-018-1568-y)
Supplement: Supplementary file 4 — Table S2. The primers used for pHIS2, pGADT7-Rec2, pCAMBIA1301, and pROKII recombinant vector construction, and qRT-PCR analysis of JrGRAS2, JrDof3 as well as 18S rRNA gene. (PDF 138 kb) [file 12870_2018_1568_MOESM4_ESM.pdf]

Table S2 The primers used for pHIS2, pGADT7-Rec2, pCAMBIA1301, and pROKII recombinant vector construction, and qRT-PCR analysis of *JrGRAS2*, *JrDof3* as well as *18S rRNA* gene.

Genes/Construct	Forward Primer	Reverse Primer
pHIS2	5’-GCCTTCGTTTATCTTGCCTGCTC-3’	5’-CGATCGGTGCGGGCCTCTTC-3’
pHIS2-DOFCOREZM	5’-AATTCAAAGAAAGAAAGGAGCT-3’	5′-CCTTTCTTTCTTTG-3’
pHIS2-DOFCOREZM-M	5’-AATTCCCCACCCACCCAGAGCT-3’	5′-CTGGGTGGGTGGGG-3’
pHIS2-DOFCOREZM-S	5’-GGAATTCCTCTGCTAAAAAGTTTTC-3’	5′-GAATCAAAAGGCGAATAGGAGCTCG-3’
pHIS2-DOFCOREZM-S-M1	5’-GGAATTCCTCTGCTAACCCATTTTC-3’	
pHIS2-DOFCOREZM-S-M2	5′-GGAATTCCTCTGCTAATTTTCAACC-3’	
pGAD-JrDof3	5′-AAGCAGTGGTATCAACGCAGAGTGGCCATTATGGCCCATGGCTGATCGAGCTCGCATGG-3’	5′-TCTAGAGGCCGAGGCGGCCGACATGTAGGGGGTTTCTGGTAGTGGAG-3’
pGAD	5’-CTATTCGATGATGAAGATACCCCACCAAACCC-3’	5’-GTGAACTTGCGGGGTTTTTCAGTATCTACG-3’
pCAM-DOFCOREZM	5’-GATCCAAAGAAAGAAAGCCCTTCCTCTATATAAGGAAGTTCATTTCATTTGGAGAGAACACGGA-3’	5’-AGCTTCCGTGTTCTCTCCAAATGAAATGAACTTCCTTATATAGAGGAAGGGCTTTCTTTCTTTG-3’
pCAM-DOFCOREZM-M	5’-GATCCCCCACCCACCCACCCTTCCTCTATATAAGGAAGTTCATTTCATTTGGAGAGAACACGGA-3’	5’-AGCTTCCGTGTTCTCTCCAAATGAAATGAACTTCCTTATATAGAGGAAGGGTGGGTGGGTGGGG-3’
pCAM-Seg	5′- GGATCCCTCTGCTAAAAAGTTTTC-3’	5′-AAGCTTCCGTGTTCTCTCCAAATGAAATGAACTTCCTTATATAGAGGAAGGGGAATCAAAAGGCGAATAG-3’
pCAM-Seg-M1	5′- GGATCCCTCTGCTAACCCATTTTC-3’	
pCAM-Seg-M2	5′- GGATCCCTCTGCTAATTTTCAACC-3’	
pCAMBIA1301	5’-TAGAGTCGACCTGCAGGCAT-3’	5’-ATCATCATCATAGACACACG-3’
pROKII	5’-TTTCATTTGGAGAGAACACG-3’	5’-TGCCAAATGTTTGAACGATC-3’
pROKII-JrDof3	5’-CTCTAGAGGATCCCC+ATGGCTGATCGAGCTCGCATGG-3’	5’- TCGAGCTCGGTACCCCTATAGGGGGTTTCTGGTAGTG -3’
pCAMBIA1301-promoter	5’-ATGCGGTACCTGAGAAAACGAAGAGATAT-3’	5’-TAGCGGATCCGATCGATCAGATAGAAAAG-3’
JrDof3(qRT-PCR)	5′-AGGTACTGGACTAGAGGT-3’	5′-TCGTGAGTGAGTTGATGC-3’
JrGRAS2(qRT-PCR)	5′-CTTGGAGCACCTCGACGAG-3′	5′-TGGAGTTGTAGGATTGCAG-3′

35S::JrGRAS2(pROKII-JrGRAS2)	5’- ATCGTCTAGAATGCTTCAGAGCTTGGTTCCAC-3’	5’- CGATGGTACCTCAGTTGGACGGCTGCCATGCC-3’
JrGRAS2-F/R	5′-ATGCTTCAGAGCTTGGTTC-3′	5′-TCAGTTGGACGGCTGCCAT-3′
18S rRNA	5′-GGTCAATCTTCTCGTTCCCTT-3′	5′-TCGCATTTCGCTACGTTCTT-3′
S-ChiP-PCR	5′-CTCTGCTAAAAAGTTTTC-3′	5′-GAATCAAAAGGCGAATAG-3′
M1-ChiP-PCR	5′-CTCTGCTAACCCATTTTC-3′	
M2-ChiP-PCR	5′-CTCTGCTAATTTTCAACC-3′	
