# Supplementary material for: The walnut transcription factor JrGRAS2 contributes to high temperature stress tolerance involving in Dof transcriptional regulation and HSP protein expression
Source: BMC Plant Biol. 2018 Dec 20;18:367. doi: 10.1186/s12870-018-1568-y (PMC6302389; doi:10.1186/s12870-018-1568-y)
Supplement: Supplementary file 5 — Table S3. The primers used for qRT-PCR analysis of HSP genes. (PDF 133 kb) [file 12870_2018_1568_MOESM5_ESM.pdf]

Table S3 The primers used for qRT-PCR analysis of *HSP* genes.

Gene	Forward primer	Reverse primer
AtHSP70B (AT1G16030.1)	5’-TGGAGATCATTCCGAACG-3’	5’-ACGACTATCATCGGCTTC-3’
AtHSP70T-1 (AT1G56410.1)	5’-GAGATCATTGCTAATGAT-3’	5’-GAGAACCATTGAAGAGAT-3’
AtHSP101 (AT1G74310.1)	5’-AGCTCATGAGCTAGCTGT-3’	5’-TCTGAGCAGCTTGAGCAC-3’
AtHsp90C (AT2G04030.1)	5’-CTCATCTGAGAAGCTCGT-3’	5’-CTTGTGACTGTATAAGCT-3’
AtHSP98.7 (AT2G25140.1)	5’-ATCACTCATGCGACTACT-3’	5’-GAATTGTCAATTCCAGCT-3’
AtHSP60-2 (AT2G33210.1)	5’-TGGAAGCAGGCTCAATTC-3’	5’-CATCACCAGCTACGTCAT-3’
AtHsp90.6 (AT3G07770.1)	5’-AGTGACACTGAATCGAGA-3’	5’-GCTGTATAAGCTGTTGAC-3’
AtHSP60-3A (AT3G13860.1)	5’-ATGTGATAATTGAGAGCAG-3’	5’-CCTGCAGCCACCGACTTG-3’
AtHSP60-3B (AT3G23990.1)	5’-ATGAGCTGGAGCAGGAAC-3’	5’-CTGCTTCACAAGACTAGC-3’
AtHSP17.4 (AT3G46230.1)	5’-CACTAGACGTATGGGATC-3’	5’-CGGTGCCATGTGTCACTC-3’
AtHSP93-III (AT3G48870.1)	5’-GTGATGAAGATGAGCAGT-3’	5’-GGTACACATCGGCTAGCT-3’
AtHSP20 (AT4G14830.1)	5’-ATCTGATCCATCACGCTC-3’	5’-TCTTGGTCACACCAGGAT-3’
AtHSP23.6 (AT4G25200.1)	5’-ACTCCTATCATCCTCCAT-3’	5’-TCCATGAACTGGTCCATC-3’
AtHSP17.6II (AT5G12020.1)	5’-TCAATCCTCGAAGACATG-3’	5’-TCTCCATCCTCACATACT-3’
AtHSP83 (AT5G52640.1)	5’-CGTGAGCTCATCAGTAAC-3’	5’-TACATCAGCTCCAGCTTG-3’
AtHsp81.4 (AT5G56000.1)	5’-AGCTGAGATCAACCAGTT-3’	5’-AATCAGCCTTGGTCATCC-3’
AtHSP21 (AT4G27670.1)	5’-CATCGAAGTCCGCTACAC-3’	5’-CTCGAACATCCTGTCCAT-3’
AtHSP18.2 (AT5G59720.1)	5’-TCGCAAGACTTATGGGAT-3’	5’-ACACGGTGCCACTTATCG-3’
Actin2 (AT3G18780)	5’-TGCAGGAGATGATGCTCC-3’	5’-ATACGAAGCTCATTGTAG-3’
JrHSP70	5’-ATGAGAGCTGCATTGTGG-3’	5’-TGCAATTCAGGATCATTG-3’
JrsHSP17.3	5’-ATGGATCTCAGAATCATG-3’	5’-CTACCTGGACCTTGATGTC-3’
JrHSP20-1	5′-CCAAGAAGAGGAAGACAC-3′	5′-TTAAGCCATCTTTGCTCT-3′
